# Molecular Evolution of the Infrared Sensory Gene TRPA1 in Snakes and Implications for Functional Studies

**DOI:** 10.1371/journal.pone.0028644

**Published:** 2011-12-07

**Authors:** Jie Geng, Dan Liang, Ke Jiang, Peng Zhang

**Affiliations:** Key Laboratory of Gene Engineering of the Ministry of Education, State Key Laboratory of Biocontrol, School of Life Sciences, Sun Yat-Sen University, Guangzhou, People's Republic of China; Smithsonian Institution National Zoological Park, United States of America

## Abstract

TRPA1 is a calcium ion channel protein recently identified as the infrared receptor in pit organ-containing snakes. Therefore, understanding the molecular evolution of TRPA1 may help to illuminate the origin of “heat vision” in snakes and reveal the molecular mechanism of infrared sensitivity for TRPA1. To this end, we sequenced the infrared sensory gene TRPA1 in 24 snake species, representing nine snake families and multiple non-snake outgroups. We found that TRPA1 is under strong positive selection in the pit-bearing snakes studied, but not in other non-pit snakes and non-snake vertebrates. As a comparison, TRPV1, a gene closely related to TRPA1, was found to be under strong purifying selection in all the species studied, with no difference in the strength of selection between pit-bearing snakes and non-pit snakes. This finding demonstrates that the adaptive evolution of TRPA1 specifically occurred within the pit-bearing snakes and may be related to the functional modification for detecting infrared radiation. In addition, by comparing the TRPA1 protein sequences, we identified 11 amino acid sites that were diverged in pit-bearing snakes but conserved in non-pit snakes and other vertebrates, 21 sites that were diverged only within pit-vipers but conserved in the remaining snakes. These specific amino acid substitutions may be potentially functional important for infrared sensing.

## Introduction

Besides their slithering body and lethal venom, perhaps the most famous feature of snakes is their extraordinary ability to sense infrared thermal radiation, also known as “heat vision”. This peculiar capability enables snakes to detect warm-blooded prey in total darkness without relying on vision, olfaction or hearing [1], [2]. Infrared detection in snakes is mediated by a specialized sensory structure called the pit organ, an exquisitely sensitive biological sensor that can detect temperature changes as small as 0.003°C [3]. Further research has shown that the pit organ may be a more general-purpose sensory system not only for prey detection, but also for environmental temperature sensation [4]. For a long time, biologists and naturalists have been trying to discover how this amazing system works. Recently, a study by Gracheva et al. [5] has revealed that a temperature-sensitive calcium ion channel, TRPA1, serves as an infrared sensory molecule in the snake pit organ, moving our understanding of this remarkable system to a molecular level.

It should be noted that not all snakes possess heat vision; only pit vipers, pythons and some boas have evolved to possess pit organs for infrared detection. Recent cellular experiments have demonstrated that snake TRPA1 channels are heat-responsive, from the pit organ–containing snakes to non-pit snakes [5]. However, the thermal thresholds of TRPA1s are quite different across snake species: ∼28°C for rattlesnakes, ∼30°C for boas, ∼33°C for pythons, and ∼37°C for non-pit rat snakes. Different thresholds are consistent with different sensitivities of these snakes toward infrared radiation [6]. Sensory systems evolve rapidly to accommodate variations in environmental niches [7]–[10]. As the core receptors of the pit organ, TRPA1 channels may have experienced particular natural selection during the history of snake evolution to fulfill functional requirements. Therefore, characterizing the evolution of TRPA1 among snakes may shed light on how snake infrared detection originated and evolved.

The study of Gracheva et al. [5] clearly revealed the TRPA1 channels as the infrared sensors in pit-bearing snakes and pointed out that the lower thermal thresholds of TRPA1s in pit-bearing snakes compared to other snakes make them more sensitive to detect changes in ambient temperature than non-pit snakes' channels. However, the genetic mechanism of such a shift of thermal thresholds is unknown. Because three different families of snakes may have independently adapted TRPA1 as an infrared sensor (based on the phylogenetic distribution of pit-bearing species), it is also intriguing to see whether and to what extent they acquired such a function through convergent evolution. To explore these questions, TRPA1 sequences of the two types of snakes (pit-bearing and non-pit-bearing) can be compared to identify amino acid changes that may be responsible for the functional alternation. Yokoyama et al. [11] recently compared four published snake TRPA1 sequences with fourteen vertebrate orthologs and identified three critical amino acids that might be involved in infrared sensitivity. Considering that this study included only a limited number of snake species, it is worthwhile to further investigate this topic using a broader taxonomic coverage of snake species.

Here, we aimed to study the molecular evolution of TRPA1 across the snake phylogeny and test whether the effect of natural selection on TRPA1s directly contributed to functional alterations in different types of snakes. In addition, by analyzing more snake TRPA1 sequences, we attempted to identify critical amino acid changes responsible for the special functions of snake TRPA1 proteins. To this end, we sequenced almost the entire coding region of the TRPA1 gene from 20 species representing many of the major lineages of extant snakes. Moreover, we specially included some non-pit snakes closely related to the three kinds of pit-bearing snakes (i.e., Viperinae snakes vs pit vipers, Xenopeltidae snakes vs pythons and non-pit boas vs pit boas). These snake species can serve as “negative controls” for the comparative study of the evolution of TRPA1 channels in the snakes, thus facilitating the identification of the critical residues.

## Results and Discussion

### Sequence Data and Phylogenetic Reconstruction

The TRPA1 calcium ion channel has six transmembrane domains with an intracellular N- and C-terminus; the N-terminus region possesses 17 ankyrin repeats [12]. In this study, we generated 20 new TRPA1 cDNA sequences. The TRPA1 sequences comprise ∼3.1 kb coding regions, including all 17 ankyrin repeats in the N-terminus and almost the entire C-terminus (lacking the final 30 bp). We also generated 19 TRPV1 cDNA sequences that contained ∼2.1 kb coding regions. The sequences that we generated spanned 93.7% and 85.3% of the protein-coding region of TRPA1 and TRPV1, respectively. In addition, because no TRPV1 sequence is available for pit boas, we analyzed the transcriptome data [5] using the rattlesnake (*Crotalus atrox*) TRPV1 as the reference sequence and assembled ∼2.4 kb TRPV1 coding sequence for the garden tree boa (*Corallus hortulanus*). Gene orthology of the newly obtained TRPA1 and TRPV1 sequences was confirmed by BlastX searches against GenBank. All sequences of the TRPA1 and TRPV1 genes determined in this paper were deposited in GenBank under accession numbers JN164328 to JN164367.

**Figure 1:**
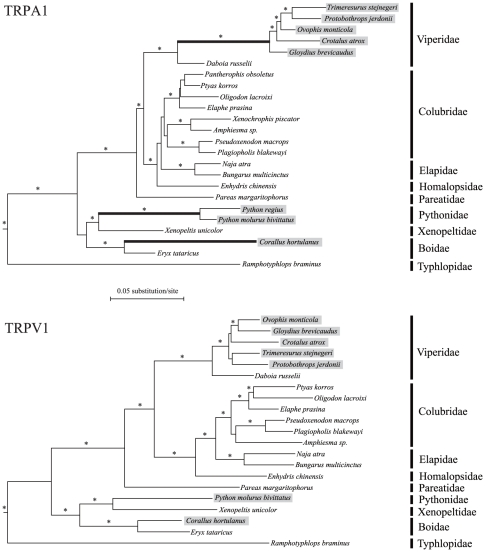
Phylogenetic relationships inferred from the TRPA1 and TRPV1 genes. Phylogenetic relationships of nine snake families were reconstructed with the ML and Bayesian methods. Non-snake outgroups are not shown. The snake species with pit organs are highlighted by shading. Branches with ML bootstrap support >70% and Bayesian posterior probability >0.95 are indicated as asterisks. Note that in the TRPA1 tree, but not in the TRPV1 tree, the three ancestral branches leading to the three groups of pit-bearing snakes (bold lines) are considerably longer than their sister branches connecting to non-pit snake relatives.

Using the ML and Bayesian methods, we reconstructed the gene trees for TRPA1 and TRPV1 at the DNA and protein levels. Both tree-building methods yielded identical tree topologies and similar branch support for a given dataset. For snakes, the phylogenetic relationships generated by the TRPA1 and TRPV1 nucleotide alignments agree with each other at the family level (Fig. 1), which are consistent with the currently accepted family-level relationships for extant snakes [13], [14]. The protein results of both genes are topologically concordant with the DNA results, but weakly supported at many nodes (results not shown). The evolution of TRPA1, the infrared sensory molecule, within snakes does not change the phylogenetic positions of the three groups of pit-bearing snakes (pit vipers, pythons and pit boas); suggesting that no apparent misleading effect of convergent evolution exists. Interestingly, we found that the length of the three ancestral branches leading to pit-bearing snakes was considerably longer in the TRPA1 tree than in the TRPV1 tree, while the remaining branch lengths show little differences between the two gene trees (Fig. 1). Thus, the acceleration of the evolutionary rate in the ancestral branches of the three groups of pit-bearing snakes is a specific event that occurred in TRPA1. This finding prompted us to postulate that the rapid evolution of TRPA1 was driven by positive selection in the lineages leading to three groups of infrared-sensitive snakes.

### Changes of Selective Pressure on TRPA1 across the Snake Phylogeny

**Figure 2:**
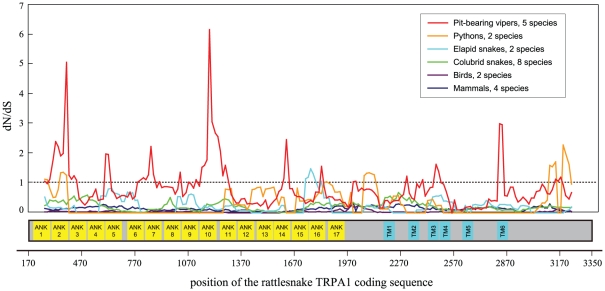
Sliding window analysis of the dN/dS ratios for six animal groups. The estimates are based on the Nei–Gojobori method. The window size was set as 150 bp, and the step size was set as 15 bp. TM, transmembrane region; ANK, ankyrin repeat.

To test whether TRPA1 is under positive selection in pit-bearing snakes, we calculated the average dN/dS ratios using the one-ratio model for the five pit vipers and two pythons. We did not calculate this ratio for pit boas because there is only one pit boa species (*Corallus hortulanus*) in our dataset. As a negative control, we also conducted the same analyses for other families of snakes without a pit organ and other non-snake vertebrates. The comparison of average dN/dS ratios showed very large differences between the two groups. The average dN/dS ratios for pit vipers and pythons were 0.820 and 0.995, respectively, while the average dN/dS ratios for colubrids (non-pit snakes), elapids (non-pit snakes), birds and mammals were only 0.230, 0.272, 0.039 and 0.167, respectively. This apparent difference can also be observed in the result of our sliding window analyses, with many peaks significantly greater than 1 for pit vipers and pythons, while the curves were uniformly below 1 for the negative control clades (Fig. 2).

The two-ratio model vs one-ratio model (2Δln L = 479.42; P<0.00001) revealed that the TRPA1 genes evolved under very different selective pressures among pit-bearing (average ω = 1.147) and non-pit snakes (average ω = 0.166), but did not provide solid evidence of positive selection. Thus, we performed site model tests to examine whether signals of positive selection were present in some sites of the pit-bearing snake TRPA1 proteins (Table 1). For pit vipers and pythons, the TRPA1 gene showed evidence for positive selection, with an estimated ω of 5.072 and 21.36 at 15.26% and 2.61% of sites across the gene, respectively. In contrast, none of the remaining datasets could reject the null model M1a, suggesting that no sites evolved under positive selection among these clades. These findings suggested that positive selection of the TRPA1 gene occurred only within the pit-bearing snakes. In addition, for pit vipers we identified nine sites as being under positive selection using the Bayes empirical Bayes method (PP>95%; Table 1). The functional significance of these particular sites is unknown, but they all fall within the N- and C-terminus and not in the transmembrane region of the TRPA1 channel, implying that the N- and C-terminus may be more responsible for infrared sensitivity.

**Table 1:** The Site Models Detecting Positive Selection in the TRPA1 Gene for Different Groups.

Data sets	N a	ln *L* (null/M1a)	ln *L* (positive selection/M2a)	2Δ (ln *L*)	*P* values	Proportion of Sites, ω>1	ω	Positively Selected Sites b
Pit vipers	5	−5630.99	−5609.33	18.66	<0.00001	0.1526	5.072	60, 218, 311, 399, 407, 410, 440, 1,030, 1,062
Pythons	2	−4614.34	−4609.16	10.36	0.0056	0.0261	21.36	—
Colubrids	8	−6564.35	−6563.55	1.60	0.4493	0.0108	3.369	—
Elapids	2	−4535.32	−4535.32	0	1	0.0632	1.000	—
Birds	2	−5056.32	−5056.32	0	1	0	1.000	—
Mammals	4	−9336.40	−9336.40	0	1	0.04711	1.000	—

a Numbers of sequences of the dataset.

b Positively selected sites identified using the Bayes Empirical Bayes method, numbered according to the full rattlesnake coding sequence. Only sites with PP>95% are presented.

**Figure 3:**
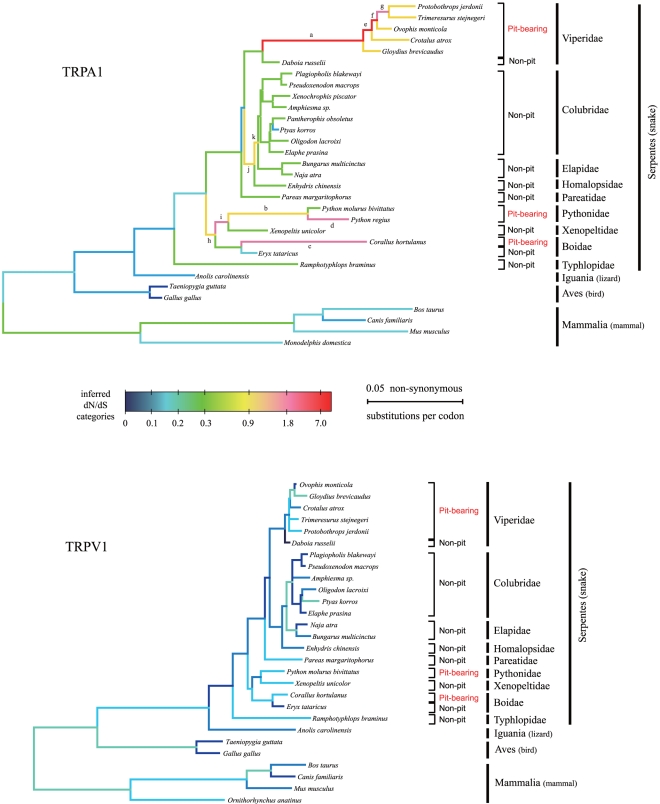
Selective pressure variation of both TRPA1 and TRPV1 along branches of the tetrapod phylogeny. The tree topology is inferred with ML for each gene. Branch lengths are drawn proportionally to the estimated numbers of nonsynonymous substitutions per codon. The trees are rooted by the frog sequence (not shown). The selective pressures of branches are assigned to dN/dS categories (tick marks along heat scale) by the GA-Branch method implemented in HyPhy. Branches assigned to a given dN/dS category share the same color. Deeper blue indicates a strong purifying selection, whereas hotter colors indicate elevated dN/dS ratios. For branches with letters along them, dN/dS values are re-estimated in PAML and subjected to a positive selection test by LRT.

The selective pressure variation among all branches of the TRPA1 and TRPV1 tree (from mammals to snakes) estimated by the GABranch method revealed more detailed information about the evolution of both genes (Fig. 3). The dN/dS ratio provides an indicator of the selective pressures that acted upon a gene over a given period with low values, indicating purifying selection and increases in values, indicating relaxation of constraint or positive selection. As a control gene, TRPV1, a closely-related TRP family member to TRPA1 but not involving in infrared sensation in snakes [5], exhibited no dramatic change in selective pressures across the tree, and the dN/dS ratios of all branches were low (0.06 to 0.19), indicating a strong purifying selection (Fig. 3). Unlike TRPV1, TRPA1 showed a great variation in selective pressures, especially within the snake lineage. For the branches belonging to mammals, birds and lizards, the dN/dS ratios remained low (0.06 to 0.3; Fig. 3), implying that the TRPA1 proteins of these animal groups may have retained a similar biological function. The dN/dS ratios within snakes increased considerably (mostly >0.3) compared to the non-snake vertebrates, indicating that the functional constraint on the TRPA1 protein became relaxed in snakes.

Another notable feature of the TRPA1 tree on molecular evolution is that the branch leading to the common ancestors of pit vipers (dN/dS = 7.071), the branch leading to the common ancestors of pythons (dN/dS = 0.976) and the branch of the pit boa *Corallus hortulanus* (dN/dS = 1.862) have much higher dN/dS values than their sister branches leading to non-pit snakes (dN/dS values 0.15∼0.3) (Fig. 3). This finding suggested that the functional alteration of snake TRPA1 proteins from infrared-insensitivity to infrared-sensitivity might be independently driven by positive selection acting on the common ancestors of pit vipers, pythons and pit boas. Besides the above three branches, there were another eight branches with dN/dS values over 0.9. Because the dN/dS values estimated by the GABranch method could not demonstrate positive selection statistically, we used branch-specific models to recalculate the dN/dS values of the eleven branches and test whether they were significantly greater than 1. We found that, except for branch K, the two-ratio model fit the dataset significantly better than the one-ratio model, but only five branches had dN/dS values significantly greater than 1 (Table 2). All five branches are ancestral branches of pit-bearing snakes or branches leading to pit-bearing snakes, indicating that positive selection of the TRPA1 gene occurred only within pit-bearing snakes.

**Table 2:** Positive Selection Detection of the Snake TRPA1 Gene along Different Branches.

Foreground branch a			Two Ratio vs. One Ratio			Two Ratio vs. Two Ratio (ω_F_ fixed = 1)			Positive selection
	ω_0_	ω_F_ (N/S) b	2Δ (ln *L*)	df^ c^	*P* values	2Δ (ln *L*)	df^ c^	*P* values	detected ?
a	0.205	6.086 (133/7)	150.64	1	<0.00001 *	27.06	1	<0.00001 *	√
b	0.210	0.647 (105/51)	34.20	1	<0.00001 *	4.28	1	0.0386 *	
c	0.204	1.523 (165/34)	119.50	1	<0.00001 *	4.02	1	0.0450 *	√
d	0.211	2.234 (54/8)	47.98	1	<0.00001 *	3.86	1	0.0495 *	√
e	0.215	Infinity (13/0)	19.26	1	<0.0001 *	5.94	1	0.0148 *	√
f	0.215	Infinity (9/0)	12.38	1	0.0004 *	4.00	1	0.0455 *	√
g	0.214	Infinity (16/0)	13.42	1	0.0002 *	0	1	1.0000	
h	0.214	Infinity (15/0)	8.62	1	0.0033 *	1.44	1	0.2301	
i	0.214	2.420 (20/3)	11.94	1	0.0005 *	0.90	1	0.3428	
j	0.215	0.696 (14/6)	4.50	1	0.0338 *	0.34	1	0.5598	
k	0.215	1.560 (5/1)	2.80	1	0.0942	0.08	1	0.7773	

a Branch labels are according to Fig. 3.

b Omega of the foreground branch (estimated numbers of nonsynonymous substitutions/estimated numbers of synonymous substitutions).

c Degrees of freedom.

*P value lower than 0.05.

Although the dN/dS value of the ancestral branch of two pythons is smaller than 1 (ω = 0.647), it is significantly higher than background branch value (Table 2). Besides positive selection, the relaxation of purifying selection due to a loss or diminishment of protein function and a reduced efficacy of purifying selection due to reduction in population size can also result in an increase in a dN/dS value. Because TRPA1 plays an important role in the infrared sensing of pythons, the first possibility of a relaxation of functional constraint can be easily ruled out. In addition, an elevated dN/dS value was not observed for the corresponding branch in the TRPV1 tree, which indicates that the second explanation is also unlikely because a shrinking population size will affect all genes. Therefore, we conclude that the elevated dN/dS value of the ancestral branch of pythons was caused by adaptive selection.

Taken together, our results clearly suggested that TRPA1 independently underwent natural selection of different degrees during the periods when the three groups of pit-bearing snakes originated. These ancient natural selection events probably enabled the TRPA1 protein to acquire an additional or modified function to serve as an infrared sensor in the ancestors of pit-bearing snakes. Then, positive selection continued during the diversification of pit vipers, pythons and pit boas, perhaps to fine-tune the thermal response properties of their TRPA1 proteins to adapt to various environmental niches.

### The Distribution of Amino Acid Substitutions Accumulated in the Ancestral Branches of Pit-bearing Snakes

Our aforementioned results demonstrated that the TRPA1s of the three groups of pit-bearing snakes (pit vipers, pythons and pit boas) accumulated more amino acid changes than their closely related non-pit relatives (Fig. 3). These accumulated changes are likely responsible for the different sensitivities to infrared stimuli between the TRPA1s of pit-bearing snakes and non-pit snakes. It is worthwhile to find out the specific domain(s) in TRPA1 that are enriched with amino acid changes in the three groups of pit-bearing snakes, as this might hint at the location of the “infrared sensory module” within the TRPA1 channel.

**Figure 4:**
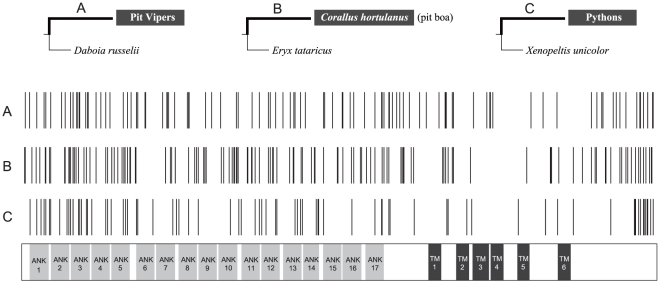
Specific amino acid substitutions (TRPA1) accumulate in three important branches. Specific amino acid substitutions (TRPA1) accumulate in branches (bold lines) leading to (A) the common ancestor of pit vipers, (B) the pit boa (*Corallus hortulanus*), and (C) the common ancestor of pythons. Specific amino acid substitutions for each branch are defined as all substitutions along the branch subtract those that also occur in the branch leading to the outgroup species (thin lines). These specific amino acid substitutions (indicated as vertical lines) are mapped above the TRPA1 domain organization schematic. Note that the substitution density of the transmembrane region (TM1 to TM6) is apparently lower than both the N-terminal region (ANK1 to TM1) and the C-terminal region (after TM6). TM, transmembrane region; ANK, ankyrin repeat.

First, we assumed that the TRPA1 channels of the last common ancestors of the three groups of pit-bearing snakes are all infrared-sensitive, while the TRPA1 channels of other non-pit relatives are not sensitive to infrared stimuli. Second, we assumed that all the amino acid substitutions that occurred in non-pit snakes are not related to the functional alteration of TRPA1 from infrared-insensitive to infrared-sensitive. Based on these two assumptions, we compared the distribution of the specific amino acid substitutions along the three ancestral branches of pit-bearing snakes (Fig. 4). The specific amino acid substitutions are defined as the residue mutations that are present in those three branches, but not in their sister branches that lead to the non-pit snake relatives (Fig. 4); these sites are probably involved in infrared sensation. Ancestral TRPA1 protein sequences were reconstructed using the CODEML program in PAML 4.4.

We identified 105, 129, and 79 specific mutation sites in the ancestral branches leading to pit vipers, pit boas and pythons, respectively. These sites were mapped to the TRPA1 domain organization schematic (Fig. 4). Overall, for all three groups of pit-bearing snakes, most of the mutation sites were interspersed in the N- and C-terminal segments, but not in the transmembrane domains. This finding suggests that the N- and C-terminal regions are candidate domains for the differences in infrared sensitivities. As a similar case, Saito et al. [15] recently reported an opposite temperature sensitivity of TRPV3 channels between mammals and western clawed frogs and also proposed the N- and C- terminal regions of the protein as the molecular determinants for the thermal difference. Additionally, in the case of TRPV2, the N- and C-terminal regions are also reported to play crucial roles in heat sensitivity in rodents [16]. In mammals, the swapping of the C-terminal regions between TRPV1 and TRPM8 channels results in an exchange of the thermal activation property [17] and gradual truncations of the C-terminal regions of the TRPV1 channel gradually alter its temperature threshold for activation [18]. Therefore, as a member of the TRP family, the C-terminal domains of TRPA1 channels may contain a domain responsible for infrared sensing. It is also noteworthy that amino acid mutations of the C-terminal region are relatively concentrated at the end of the domain, but amino acid mutations of the N-terminal region were distributed more evenly and not enriched in any ankyrin motifs (Fig. 4). In the future, it will be necessary to perform “swapping” experiments for both terminals to understand the molecular basis for the differences in the infrared sensitivities of TRPA1 channels between pit-bearing and non-pit snakes.

### Amino Acid Sites of Potentially Functional Importance for Infrared Sensing

**Figure 5:**
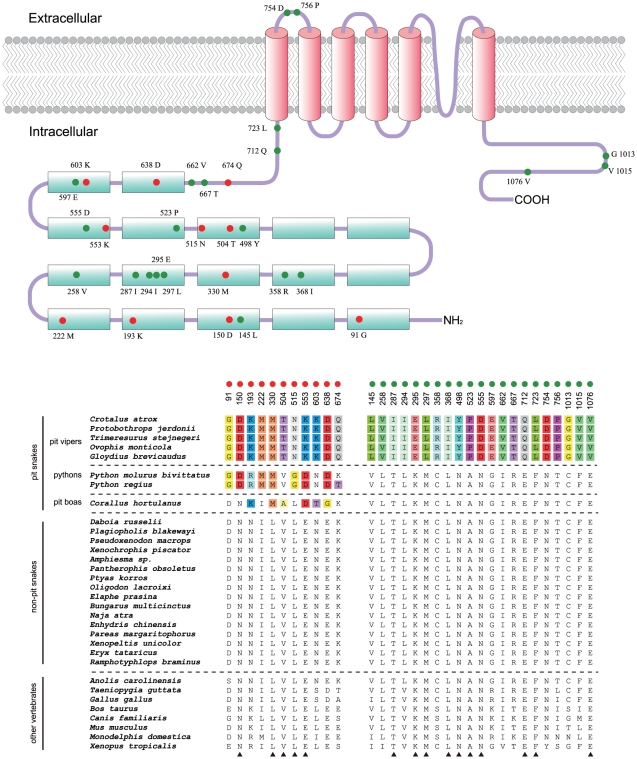
Amino acid sites of the TRPA1 protein that may be functionally important for infrared detection. Amino acid residues that are highly conserved in all non-pit snakes, but divergent in the other three groups of pit-bearing snakes, are indicated by red circles. Amino acid residues that are separately conserved within pit vipers and the remaining snakes are indicated by green circles. These sites are numbered according to the full rattlesnake TRPA1 protein sequence. Positions conserved across all non-pit snakes and other non-snake vertebrates are indicated by black triangles below the sequences. These functionally important sites are mapped to the TRPA1 channel functional schematic (cyan blocks represent ankyrin repeats, and pink cylinders represent transmembrane domains). Note that most sites are distributed in the N-terminal domain (ANK1 to TM1), rather than the C-terminal domain (after TM6), suggesting that the N-terminal domain may be the responsible region for infrared detection capacity.

Parallel amino acid replacements responsible for parallel functional changes have been reported many times [10], [19]–[23]. Because three groups of snakes (pit vipers, pythons and pit boas) independently gained the ability to sense infrared radiation, the sensory TRPA1 channels are thought to contain some convergent amino acid changes among the three groups of infrared-sensitive snakes. Yokoyama et al. [5] recently identified three parallel amino acid replacements (L330M, Q391H, and S434T) that may be critical for the development of infrared vision in the three groups of snakes. Here, we wanted to test whether the three predicted sites are still reliable when more snake TRPA1 sequences are analyzed. We first generated a protein alignment of TRPA1 for all snake and non-snake species used in this study (Figure S1). Following the criterion used by Yokoyama et al. [11], we searched for amino acids that are common in all the pit-bearing snakes, but not other non-pit snakes and non-snake vertebrates. Of the three previously predicted sites, only one site (L330M; Fig. 5) still met the criterion, while the other two sites showed no convergence for the pit-bearing snakes (for details, see Figure S1).

The study of Gracheva et al. [5] showed that the thermal thresholds of TRPA1s in pit-bearing snakes are lower than those in other non-pit snakes and this may be the molecular basis of infrared sensitivities of pit-bearing snakes. Although the shift of thermal thresholds may be caused by the single amino acid substitution (L330M), other amino acid replacements contributing to the lower thermal threshold of TRPA1 in pit-bearing snakes may be involved for the following reasons: (1) it is unlikely that only one substitution is responsible when TRPA1 channels in pit-bearing snakes have accumulated so many specific amino acid changes (Fig. 4); and (2) the thermal thresholds of TRPA1s in pit vipers, pythons and pit boas are different, so the functionally important sites may also diverge to different states in the three groups of snakes. Accordingly, we modified our search criterion to seek for sites that diverge to different states in pit-bearing snakes (target group) but remain invariable in all other non-pit snakes (control group). Because sites that remain constant in all non-pit snakes are likely to be functionally important, substitutions at these sites would be expected to result in the relaxation of functional constraint or functional modifications; where this has occurred in pit-bearing snakes, it would imply that the corresponding sites are involved in the decrease of the thermal thresholds (an important step towards infrared sensitivity). It must be noted that our functional inference is based on an indirect relation between a signal trait (infrared sensitivity) and the substitution observations, and could be affected by many other traits such as habitat, seasonality, preferred thermal niche. Therefore, the functional inference is just putative and should be tested by future experiments.

We found eleven sites (positions 91, 150, 193, 222, 330, 504, 515, 553, 603, 638, and 674; numbered according to the full rattlesnake TRPA1 protein sequence) met this criterion (red circles; Fig. 5). Of these sites, five (positions 150, 330, 504, 515, and 553) are conserved not only within all non-pit snakes, but also within other non-snake vertebrates. Notably, all eleven identified sites are located within the N-terminal region, suggesting that the N-terminal region may play a main role in thermal sensing. Because the thermal thresholds of TRPA1s of pit vipers (∼28°C) are lower than those of pythons (∼33°C) and pit boas (∼30°C) [5], the sites specifically altered within the pit vipers, but retained invariable in all the remaining snakes, may contribute to further turning down the thermal threshold of the protein (enhance the infrared sensitivity). Thus, we set the pit vipers as the target group and all the other snakes as the control group, and we repeated the seeking procedure. As a result, 21 amino acid changes (positions 145, 258, 287, 294, 295, 297, 358, 368, 498, 523, 555, 597, 662, 667, 712, 723, 754, 756, 1013, 1015, and 1076) were identified (green circles; Fig. 5). Of these sites, 16 are distributed across the N-terminal region, 2 are within the transmembrane region, and 3 are within the C-terminal region (Fig. 5). This result suggests that the enhanced infrared sensitivity of the pit viper TRPA1 channels may be more related to the modifications within the N-terminal region.

The N-terminal region of the TRPA1 protein comprises 17 ankyrin repeats, which are common structural motifs that mediate protein–protein interactions [24]. Previous research indicates that mutations in the ankyrin repeat domain of TRPV1, TRPV3, and TRPV4 influences their binding profiles with ATP and calmodulin, further altered the thermosensitive properties of these proteins [25]. Given that most of the infrared-related sites identified in this study are located within the ankyrin repeat domain of TRPA1, it is possible that these substitutions allow the TRPA1 channels of pit-bearing snakes to interact with a specific protein or ligand that enables the infrared sensitivity. However, we could not rule out the possibility that the ankyrin repeat domain of TRPA1s in pit-bearing snakes function as the thermal sensor. Long ankyrin repeats of TRPA1 have been thought to act as molecular springs to sense mechanical stimuli [26], and the thermal stimulation generated by infrared radiation is a type of mechanical stimuli at the molecular level. Therefore, the substitutions in ankyrin repeats may change the elastic properties of the pit-bearing snake ankyrins, thus enabling them to respond to the thermal stimuli generated by infrared radiation.

## Materials and Methods

### Taxon Sampling and Experimental Procedures

To better understand TRPA1 gene evolution across the snake phylogeny, we collected 20 snake species in the field or from private breeders. These 20 snake species, together with the 4 snakes that already have published TRPA1 sequences, represent 9 families of extant snakes spanning a broad evolutionary coverage (Viperidae, Pythonidae, Boidae, Colubridae, Elapidae, Homalopsidae, Pareatidae, Xenopeltidae, and Typhlopidae). Moreover, one lizard, two birds, five mammals and one frog were used as outgroup taxa in the phylogenetic and evolutionary analyses. Detailed information for all species used in this study is listed in Table 3. This study was performed in strict accordance with the guidelines developed by the China Council on Animal Care and Use. All animal processing procedures were approved by the Institutional Animal Care and Use Committee of Sun Yat-Sen University (permit number: 2010–034).

**Table 3:** The species used in this study; newly generated sequences are in bold.

Taxonomy	Species	Collection locality	Withpit organ	Accession number	
				TRPA1	TRPV1
Typhlopidae	*Ramphotyphlops braminus*	Hongkong, China	-	**JN164334**	**JN164355**
Pythonidae	*Python regius*	----	yes	GU562965	
	*Python molurus bivittatus*	Private breeding	yes	**JN164338**	**JN164363**
Xenopeltidae	*Xenopeltis unicolor*	Mangshan, Hunan, China	-	**JN164340**	**JN164360**
Boidae	*Corallus hortulanus*	----	yes	GU562969	**JN164348** *
	*Eryx tataricus*	Private breeding	-	**JN164342**	**JN164362**
Pareatidae	*Pareas margaritophorus*	Bawanglin, Hainan, China	-	**JN164329**	**JN164350**
Homalopsidae	*Enhydris chinensis*	Shaoguan, Guangdong, China	-	**JN164347**	**JN164367**
Viperidae	*Daboia russelii siamensis*	Shaoguan, Guangdong, China	-	**JN164343**	**JN164364**
	*Ovophis monticola*	Mengzhi, Yunnan, China	yes	**JN164341**	**JN164361**
	*Protobothrops jerdonii*	Tianquan, Sichuan, China	yes	**JN164328**	**JN164349**
	*Gloydius brevicaudus*	Shaoguan, Guangdong, China	yes	**JN164330**	**JN164351**
	*Crotalus atrox*	----	yes	GU562967	GU562968
	*Trimeresurus stejnegeri*	Yongzhou, Hunan, China	yes	**JN164332**	**JN164353**
Elapidae	*Naja atra*	Shaoguan, Guangdong, China	-	**JN164339**	**JN164359**
	*Bungarus multicinctus*	Shaoguan, Guangdong, China	-	**JN164345**	**JN164366**
Colubridae	*Ptyas korros*	Shaoguan, Guangdong, China	-	**JN164335**	**JN164356**
	*Xenochrophis piscator*	Shaoguan, Guangdong, China	-	**JN164346**	
	*Elaphe prasina*	Mengzhi, Yunnan, China	-	**JN164337**	**JN164358**
	*Pseudoxenodon macrops*	Mengzhi, Yunnan, China	-	**JN164331**	**JN164352**
	*Oligodon lacroixi*	Mengzhi, Yunnan, China	-	**JN164344**	**JN164365**
	*Plagiopholis blakewayi boulenger*	Mengzhi, Yunnan, China	-	**JN164336**	**JN164357**
	*Pantherophis obsoletus lindheimeri*	----	-	GU562966	
	*Amphiesma sp.*	Mengzhi, Yunnan, China	-	**JN164333**	**JN164354**
Iguania	*Anolis carolinensis*	----	-	ENSACAT00000014581	ENSACAT00000014065
Aves	*Gallus gallus*	----	-	ENSGALT00000025203	NM_204572
	*Taeniopygia guttata*	----	-	XM_002197822	XM_002195904
Mammalia	*Mus musculus*	----	-	NM_177781	NM_001001445
	*Canis familiaris*	----	-	XM_544123	NM_001003970
	*Bos taurus*	----	-	XM_581588	XM_002695751
	*Monodelphis domestica*	----	-	ENSMODT00000009119	
	*Ornithorhynchus anatinus*	----	-		ENSOANT00000020090
Amphibia	*Xenopus tropicalis*	----	-	NM_001127962	ENSXETG00000005790

*assembled from the transcriptome data[5] using the rattlesnake (*Crotalus atrox*) TRPV1 as the reference.

Nerve tissue samples (brain, trigeminal ganglia, spinal nerve, etc.) were preserved in RNAlater (Ambion) in the field, shipped to the laboratory within a week and stored at −80°C. Total cellular RNA was extracted from about 20 mg of the preserved tissue mixture using the RNA prep pure tissue kit (Tiangen, Beijing) and then reverse transcribed into cDNA by primeScript RTase (Takara, Dalian) with dT_25_. Based on the currently available TRPA1 and TRPV1 mRNA sequences of snakes, lizards and birds (Table 3), 9 PCR primers (Table 4) were designed to amplify the target TRPA1 and TRPV1 cDNA fragments using a nested PCR strategy. For the TRPA1 fragment, the first step PCR mixture (25 µl total) contained 1 µl of RT template, 2.5 µl of 10× buffer, 1 µl of primer AF1, 1 µl of primer AR, and 1 U of High-Fidelity DNA polymerase (TransGen, Beijing), and PCR was conducted using the following cycling settings: 4 min at 94°C of initial denaturing; 30 cycles of denaturing at 94°C for 45 s, annealing at 50°C for 40 s, extending at 72°C for 3.5 min; and a final extending step of 72°C for 10 min. The second step PCR was conducted using the same procedure but with 1 µl of the first step PCR product as a template and AF2 and AR as primer pairs. For the blind snake *Ramphotyphlops braminus*, AF2 and AR did not work; thus, the primers AF2-1 and AR-1 were used instead. Target bands were purified by agarose gel extraction (Tiangen, Beijing) and cloned into a PMD19-T vector (Takara, Dalian). For each of the cloned bands, at least three positive recombinant clones were identified by colony PCR and sequenced on an automated ABI3730 DNA sequencer. The TRPV1 fragments were amplified and sequenced using a similar methodology.

**Table 4:** The primers used to amplify the TRPA1 and TRPV1 cDNA fragments.

Target gene	Forward primer(5′→3′)	Reverse primer((5′→3′)
TRPA1	AF1: GCCTCGCAGGATRTCTTYAARGT	AR: CTTTSARSACYGTRTCCCAYTT
	AF2: CTYCGRAGYTTYATHAARAARAA	AR-1: TCTCCATCTGCTGCTTTCTG
	AF2-1: TACTTGCCGACTTCGAAGCCTTA	
TRPV1	VF1: GAYGAYCACACSGAYGGSGARGA	VR1: GGNACCAANGTYTTCCARTTYTT
	VF2: AARGAYATGGCTCCNATGGAYTC	VR2: CCSGGRTCYTCRTTGATDAT

### Phylogenetic Reconstruction and Evolution Analyses

Deduced amino acid sequences of TRPA1 and TRPV1 were aligned with CLUSTALW [27] and modified with BioEdit [28]. The nucleotide alignments were generated according to amino acid alignments. The best-fitting models for the TRPA1 and TRPV1 DNA alignments were separately selected by AIC and implemented in MrModelTest2.3 [29]. The GTR+I+Γ model was chosen as the best-fitting model for both the TRPA1 and the TRPV1 genes. ML analyses were implemented using RAxML 7.0.3 [30] with 1000 rapid bootstrap replicates. Bayesian analyses were performed in MrBayes 3.1.2 [31]. Two MCMC runs were performed with one cold and three heated chains (temperature set to 0.2) for 10 million generations and sampled every 500 generations. The first 25% of sampled trees were discarded as the burn-in. Similar topologies and posterior clade probabilities from the two runs were observed. We also analyzed the protein alignments of TRPA1 and TRPV1 following the same methodology using the JTT+Γ as the amino acid substitution model.

Because TRPA1 is the receptor molecule in the pit organ, we suspect it may experience natural selection within pit-bearing snakes. To visualize selective variation among the different amino acid sites of the TRPA1 gene, we performed a sliding window analysis using the program SWAAP 1.0.2 [32], comparing pit-bearing snakes with other non-pit snakes and vertebrates. The window size and the step size were set at 150 bp and 15 bp, respectively. Values of ω were estimated following Nei and Gojobori [33]. To test for evidence of positive selection in TRPA1 across certain groups, we implemented site models with the CODEML program in PAML 4.4 [34], comparing Model M1a with Model M2a using the LRT test. We also applied a two ratio model test with CODEML to the entire TRPA1 dataset, with one omega parameter assigned to all pit-bearing snake clades and the other assigned to the remaining lineages (non-pit snakes and other vertebrates), to detect if the TRPA1gene of all pit-bearing snake species experienced divergent patterns of selection compared to non-pit species and other vertebrates.

After finding evidence of significant variation in ω of TRPA1 across different groups, we applied the GABranch method [35] to investigate how this variation was distributed across the branches of the TRPA1 tree. This analysis was conducted using downloadable HyPhy script (http://www.hyphy.org/gabranch/) implemented in HyPhy version 1.0 [36]. The nucleotide model was specified as GTR; otherwise, the default GABranch configuration was used. Unlike the free-ratio model implemented in PAML, the GABranch method does not calculate the ω-value precisely for a given branch but assigns it to a ω-category, avoiding the overparameterization problem of the free-ratio model (PAML manual) [34]. Although the GABranch method is useful to assign branches into similar selective categories, the estimated ω-value is not ideal. Therefore, for those branches assigned to ω-categories exceeding (or nearly exceeding) one by the GABranch method, we used the branch models in PAML 4.4 to recalculate the ω-value and tested whether the ω-value of a given branch was significantly higher than one (evidence for positive selection). In addition, as a “negative control”, we also applied the GABranch analysis to the TRPV1 gene. Because TRPV1 belongs to the TRP channel family, like TRPA1, but is not involved in infrared detection [5], we had expected to observe a different pattern of selective pressure variation across branches of the TRPV1 tree.

In order to identify amino acid changes that may be responsible for infrared sensitivity in pit-bearing snake TRPA1 proteins, we adopted the function-based method used by Yokoyama et al. [11]. This method aims to identify amino acid residues that are totally conserved in the “control group” (functionally important) but diverged to different states in the “target group” (functional divergence). In this case, the control group is composed of the TRPA1 proteins of all sampled non-pit snakes, which we assume are not infrared-sensitive; the target group is the TRPA1 proteins of all sampled pit-bearing snakes. Because the infrared sensitivity of pit vipers is 5–10-fold higher than pythons or boas [6], we also set the pit vipers as the target group, while all other snakes were set as the control group, to identify amino acid changes that may contribute to further enhancing infrared sensitivity.

## Supporting Information

Figure S1 **Protein Sequence Alignment of TRPA1 including Snakes, Lizards, Birds, Mammals, and Amphibians.** Putative ankyrin repeats (ANK) and transmembrane domains (TM) are indicated by black squares. The three amino acid sites proposed by yokoyama et al. (2011) that seem convergent in pit-bearing snakes are indicated by blue arrows.
(PDF)
