# Supplementary material for: Molecular Evolution of the Infrared Sensory Gene TRPA1 in Snakes and Implications for Functional Studies
Source: PLoS One. 2011 Dec 7;6(12):e28644. doi: 10.1371/journal.pone.0028644 (PMC3233596; doi:10.1371/journal.pone.0028644)
Supplement: Figure S1 — Protein Sequence Alignment of TRPA1 including Snakes, Lizards, Birds, Mammals, and Amphibians. Putative ankyrin repeats (ANK) and transmembrane domains (TM) are indicated by black squares. The three amino acid sites proposed by yokoyama et al. (2011) that seem convergent in pit-bearing snakes are indicated by blue arrows. (PDF) [file pone.0028644.s001.pdf]

ANK1 ANK2

```
                                   10        20        30        40        50        60        70        80
                          ....|....|....|....|....|....|....|....|....|....|....|....|....|....|....|....|
Crotalus atrox            NRRRLKKLDRLNATPLHHAAGNGQLELMQMIMDGSSPEALNVADVSGNTPLHWATKKHQTESIKLLLSRGANPNILNVNM
Protobothrops jerdonii    NRRGLKKLDRMNATPLHHAAGNGQLELMQMIMDGSSPQALNVTDVSGNTPLHWATKKQQTESIKLPLSKGADPNILNVNL
Trimeresurus stejnegeri   NRRGLKKLDRLNATPLHHAAGNGQLELMQMIMDGSSPEALNVTDVTGNTPLHWATKKQQTESIKLLLSKGANPNILNVNL
Ovophis monticola         NRSGLKKLDRLNATPLHHAAGSGQLELMQMIMDGSSPEALNVTDVTGNTPLHWATKKQQTESIKLLLSKGANPNILNVNL
Gloydius brevicaudus      NRSGLKKLDRLNATPLHHAAGSGQLELMQMIMDGSSPEALNATDVSGNTPLHWATKKQQTESIKLLLSKGANPNILNVNM
Python regius             NRNGLKKLDEMKATPLHHAAGGGQLELMLMIMDGSSAEALNIGDAYGNTPLHWATKKHQIESVKLLLSRGASPNSLNKNN
Python molurus bivittatus NRNGLKKVDEMNATPLHHAAGGGQLELMLMIMDGSSAEALNVADAYGNTPLHWATKKHQIESVKLLLSRGASPNTLNKNM
Corallus hortulanus       NSKGLKKLDDMNASPLHHAAANGQLEFMQMIMDDSPPEALNVADNAGNTPLHWATKKQQIESVKLLLRKGANPNVANANM
Xenopeltis unicolor       NRNGLKKLDELNATPLHHAAGNGQLELMQMIMDDSSVEALNVTDSCGNTPLHWATKKHQTESIKLLLSRGANPNILNYNM
Eryx tataricus            NRNGLKKVDELNATPLHHAAGNGQLELMQMILDDCSVEALNVTDSVGNTPLHWATKKQQIESVKLLLSRGANPNILNSNL
Daboia russelii           NREGLKKVDQLNATPLHHAAGKGQLELMQMIMDDSSFEALNVTDSSGNTPLHWATKKQQTESVKLLLSRGANPNILNSNM
Pantherophis obsoletus    NREGLKKLDKLNATPLHHAAGKGQLELMQMIMDDSSFEALNVTDSSGNTPLHWATKKQQTESVKLLLSRGANPNILNSNM
Ptyas korros              NREGLKKLDQLNATPLHHAAGRGQLELMQMIMDDSSFEALNATDSSGNTPLHWATKKQQTESVKLLLSRGANPNILNSNM
Oligodon lacroixi         NREGLKKLDQLNATPLHHAAGRGQLELMQMIMDDSSFEALNVTDSSGNTPLHWATKKQQTESVKLLLSRGAKPNILNSNM
Elaphe prasina            NREGLKKLDPLNATPLHHAAGSGQLELMQMIMDDSSFEALNVTDSSGNTPLHWATKKQQTESVKLLLSRGANPNILNSNM
Pseudoxenodon macrops     NREGLKKVDQLNATPLHHAAGRGQLDLMQMIIDDSSFEALNVADSSGNTPLHWATKEQQTESVKLLLSRGANPNILNSNM
Amphiesma sp.             NREGLKKLDQLNATPLHHAAGRGQLELMQMVMDDSSFEALNVTDSSGNTPLHWATKKQQIESVKLLLSRGANPNILNSNM
Xenochrophis piscator     NREGLKKLDQLNATPLHHAAGKGQLELMQMIMDDASFEVLNVTDSSGNTPLHWATKKQQTESVKLLLSRGANPNILNSNM
Plagiopholis blakewayi    NREGLKKLDKLNATPLHHAAGRGQLDLMQMIMDDSSFEALNVADSSGNTPLHWATKEQQPESVKLLLSRGANPNILNSNM
Naja atra                 ??????????????????????????LMQMIMDDSSFEALNVTDSSGNTPLHRATKEQQTESVKLLLSRGANPNILNSNM
Bungarus multicinctus     NREGLKKVDQQNATPLHHAAGRGQLELMQMILDDSTFEALNVTDSSGNTPLHWATKEQQTESVKLLLSRGANPNILNSNM
Enhydris chinensis        NREGLKKLDQLNATPLHHAAGKGQLELMQVIMDDSSFEALNVTDSSGNTPLHWATKEQQIESVKLLLSRGANPNILNSNM
Pareas margaritophorus    NREGLKKLDQLNATPLHHAAGKGQLELMQMIIDDSSFEALNVTDSSGNTPLHWATKKQQTESVKLLLSRGANPNILNSNM
Ramphotyphlops braminus   NRDGLKKVDELNATPLHHAAGGGQLELMQVIIDDSSVEALNVADLYGNTPLHWATTKHQPESVKLLLSRVASPNILNSSM
Anolis carolinensis       NREGLKKLDDLNATPLHHAAGQGQLELMQMIIDSSSE-ALNVTDTSGNTPLHWATKKNQIESVKLLLSRGANPNILNSNM
Taeniopygia guttata       NRSGLTKVDELNATPLHHAAEGGQIELMQLIIDDSSCEVLNVMDSSGNTPLHWATKKNQVESVRLLLSRGANPNILNSNM
Gallus gallus             NRSGLTKVDELNATPLHHAAEGGQIELMQLIIDDSSSEVLNVMDSSGNTPLHWATRKNQVESVRLLLSRGANPNILNSNM
Bos taurus                QK-RFKKYDDVNASPLHHAAEEGQVELMEMIVNESSCEVLNVMDDYGNTPLHWAAGKNQVESVKFLLRKGANPNLRNCSM
Mus musculus              RR-KLSKYEDENLCPLHHAAAEGQVELMELIINGSSCEVLNIMDGYGNTPLHCAAEKNQVESVKFLLSQGANPNLRNRNM
Canis familiaris          RK-KLNKYDNMNTSPLHHAAEEGQVELMEMIISDSSFEVLNVMDDYGNTPLHWAAEKNQVESVKFLLSKGANPNLRNSNM
Monodelphis domestica     NRDCLGTYDELNATPLHHAAAQDQVELLEMIIDDSSLEGLNIMDHFGNTPLHWATEKKQVENVKLLLRRGADPNICNNNL
Xenopus tropicalis        NPGSLAARDDSEATPLHHAAKLGNIDLMLMIVNESAEEGINALDSRGNTPLHWAVQKNKLDCLKVLLCRGADPNIQNCYR

                                   90       100       110       120       130       140       150       160
                          ....|....|....|....|....|....|....|....|....|....|....|....|....|....|....|....|
Crotalus atrox            IAPLHWALHYLLDDLVTIFLECSNTNINLEGEGGNTPILLACYKDNPTALKILIEKGGDICKVNNMGCMPVHAAAFSGSK
Protobothrops jerdonii    IAPLHWALHYLLDDLVKIFLECNNTNINLEGEGGNTPILLACYKDNPDALKLLIEKGGDICKVNNMGCMPVHAAAFSGSK
Trimeresurus stejnegeri   IAPLHWALHYLLDDLVKIFLECNNTNINLEGEGGNTPILLACYKDNPDALKLLIEKGGDICKVNNMGCMPVHASAFSGSK
Ovophis monticola         IAPLHWALHYLLDDLVKIFLECNNTNINLEGEGGNTPILLACYKDNPDALKLLIEKGGDICKVNNMGCMPVHAAAFSGSK
Gloydius brevicaudus      IAPLHWALHYLLDDLVKIFLECNNTNINLEGEGGNTPILLACYKDNPDALKLLIDKGGDICKVNNMGCMPVHAAAFSGSK
Python regius             MAPLHWAVQYFFDDLLKIFIENDMTEINLEGESGNTAILLACYKDNPEALKLLIERGADICKANHMGTMPIHAAAFSGSK
Python molurus bivittatus MAPLHWAVQYFFDDLLKIFLENSMTEINLEGESGNTPILLACYKDNPEALKLLIERGGDICKANHMGCMPVHAAAFSGSK
Corallus hortulanus       MTPFHWVVQYHFNDLVKIFLENSLTDVNLGGEGGNTPILLACYKDNPEALKLLIEKGGDICKANNTGCMAVHAAATSGAK
Xenopeltis unicolor       MSPFHWAVQYLFNDLVKIFIENSTTDINLEGEGGNTPILLACYKDNPEALKLLIENGGEICKANNMGCMPVHAAAFSGAK
Eryx tataricus            MSPLHWAVQYLFNDLVKIFIENSTTDVNLEGEGGNTPILVACYKDNPEALKLLIENGGEICKANNMGCMPVHAAAFSGAK
Daboia russelii           ISPLHWAVLYLLNDLVKIFLGCSNTDVNLEGEGGNTPILIACYKDNPEALKLLIENGGDICKANNMGCMPVHAAAFSGSK
Pantherophis obsoletus    VSPLHWAVQYLCNDLVKIFLECSITDVNLEGEGGNTPILVACYKDNSEALKLLIENGGDIAKANNMGCMPVHAAAFSGSK
Ptyas korros              VSPLHWAVLYLCNDLVKIFLECSTTDVNLEGEGGNTPILVACYKDNSEALKLLIENGGDIAKANNMGCMPVHAAAFSGSK
Oligodon lacroixi         VSPLHWAVLYHCNDLVKIFLECSTTDVNLEGEGGNTPILLACYKDNSEALKLLIENGGDITKGNNMGCMPVHAAAFSGSK
Elaphe prasina            VSPLHWAVMYLFNDLVQIFLECSITDVNLEGEGGNTPILVACYKDNSEALKLLIENGGDIAKANNMGCMPVHAAAFSGSK
Pseudoxenodon macrops     ISPLHWAVLYLFNDLVKIFLECSTTDVNLEGEGGNTPILGACYKDNSEALKLLIENGGDIAKANNMGCMPVHAAAFSGSK
Amphiesma sp.             ISPLHWAVLYLLNDLVKIFLECSTTDVNLEGESGNTPIIVACYKDNPEALKLLIENGGDIAKANNMGCMPVHAAAFSGSK
Xenochrophis piscator     ISPLHWAVLYLLNDLVKIFVECSATDVNLEGEGGNTPILVACYKDNHEALKLLIENGGDIAKANNMGCMPVHAAAFSGSK
Plagiopholis blakewayi    ISPLHWAVLYLFNDLVKIFLECSTTDVNLEGEGGNTPILGACCKDNSEALKLLIENGGDISKANNMGCMPVHAAAFSGSK
Naja atra                 ISPLHWAVLYLFNDLVKIFLECSTVDVNLEGEAGNTPILVACYKDNAEALKLLIENGGDIVRANNMGCMPVHAAAFSGSK
Bungarus multicinctus     ISPLHCAVLYLFNDLVKIFLECSTVDVNLEGEAGNTPILVACYKDNAEALKLLIENGGDIAKANNMGCMPVHAAAFSGSK
Enhydris chinensis        ISPLHWAVLYLLNDLVKIFLEYSITDVNLEGESGNTPILIACCKDNSEALKLLIENGGDITKANNMGCMPVHVAAFSGSK
Pareas margaritophorus    VSPLHYAVLYLFNDLVKIFLECSSTDVNLVGEGGNTSILAACYKDNPEALKLLIENGGEICRANNMGCMPVHAAAFSGSK
Ramphotyphlops braminus   MAPLHLAVQYHYNDLVQIFTEHSTTNVNLEGESGNTPVLVACYKDNSEALKLLLENGGQINKPNNIGCMSVHAAAFSGSK
Anolis carolinensis       MAPLHLAVQSLRNELVKIFVEHTTTDINLEGENGNTPITIACYKDNSEALKLLIENGAKICKGNSMGWMPVHAAAFSGAK
Taeniopygia guttata       MAPLHMAVQSLHNEIVKVLVQHSSTDVNLEGEAGNTPIIVACYKDNPEALTLLVENGGKICKPNKTGCMPIHAAAFSGAK
Gallus gallus             MAPLHMAIQSLHNEIVKILVQHSSTDVNLEGEAGNTPIIVACYKDNPEALTFLIENGGKICKPNKTGCMPIHAAAFSGAK
Bos taurus                MAPLHVAVQGGYNDVMKVLIEHSSTDVNLEGENGNTALIITCFTDNSEAMQLLLNKGAKPCKSNKWGCFPIHQAAFSGAK
Mus musculus              MSPLHIAVHGMYNEVIKVLTEHKATNINLEGENGNTALMSTCAKDNSEALQILLEKGAKLCKSNKWGDYPVHQAAFSGAK
Canis familiaris          MAPLHIAVQGLHNEVIKVLTEHNSTDINLEGENGNTAVIIACLKDNSEALQILLNKGAKPCKSNKWGCFPIHQAAFSGAK
Monodelphis domestica     MAPLHLAIQSCYNEIVKVLVEHSSTDVNLKGENGSTPVLLACSKDNSEALKLLMDRGAKICRPNKFGCYPVHIATFSGAK
Xenopus tropicalis        LSPLHLAIQLFHNSIAEALILHSTTDINLEGDLGNTPVMQACCKDNHEALNLLLGRGAKLCKQNKLGCYPIHMTAFMGSL
```

ANK3 ANK4 ANK5

ANK5 ANK6 ANK7

```
                              170          180          190          200          210          220          230          240
                          ....|....|....|....|....|....|....|....|....|....|....|....|....|....|....|....|
Crotalus atrox            LCLEMIIKRGEQLGYSPKNHINFINNEKSSPLHLAVQSRDVEMIKMCIEYGAQIDLKQSDNCTALHIAAIQGATEIIELL
Protobothrops jerdonii    LCLEMIIKRGEQLGYLPKNHINFINNEKSSPLHLAVQSRDVDMIKMCIEYGAQIDLKQGDNCTALHFAAIQGATEIIELL
Trimeresurus stejnegeri   VCLEMIIKRGEELGYLPKNHINFINNEKSSPLHLAVQSRDVDMIKMCLEYGAQVDLKQSDNCTALHYAAIQGATEIIELL
Ovophis monticola         VCLEMIIKRGEQLGYSPKNHINFINNEKTSPLHLAVQSRDVDMIKMCIEFGAQIDLKQSDNCTALHFAAIQGATEIIELL
Gloydius brevicaudus      SCLEMIIKRGEQLGYSPKNHINFINNEKSSPLHLAVQSRDVDMIKMCIDYGAQIDLKQNDNCTALHFAAIQGATEIIELL
Python regius             ICMEMVIKQAEKLGHSPESHINSLDNARNSPLHLAVQSRDLEMIRMCIEYGAQVDLKQNDKCTALHFAATQGATEILKLM
Python molurus bivittatus SCLEMIIKQGEKLGHSPESHINFTNNAKSSPLHLAVQSRDLEMIKMCIEYGAQVDLKQNDKCTALHFAATQGATEILKLM
Corallus hortulanus       LCMEIVIRRGEDLGYTRESHINFTTTGKSSPLHLAVQSRDLEMIKMCIEYGAQIDLKQNDNCTALHFAATQGATDILKLM
Xenopeltis unicolor       LCLEIIIKRGEELGHSPESHINFTNNGKSSPLHLAVQSRDLEMIKMCIEYGAQIDLKQNDKCTALHFAATQGATEILKLM
Eryx tataricus            LCLEIIIKRGEELGYSPESHINFTNNGKSTPLHLAVQSRDLEMIKMCIEYGAQIDLKQNDKCTALHFAATQGATEILKLM
Daboia russelii           LCLEIIIKRGEELGYSPENHINFINNGKCSPLHLAVQSRDLEMIKMCIEYGAKIDLKQSDNCTALHFAATQGATEILKLM
Pantherophis obsoletus    LCLEIIIKRGVELGYSPENHINFTNNGKCSPLHLAVQSRDLEMIKMCIEYGAQIDLKQNDNCTALHFAATQGATEILKLM
Ptyas korros              LCLEIIIKRGVELGYSPENHINFTNNGKCSPLHLAVQSRDLEMIKMCIEYGAQIDLKQNDNCTALHFAATQGATEILKLM
Oligodon lacroixi         LCLEIIIKRGEELGYSPENHINFTNNGKCSPLHLAVQSRDLEMIKMCIEYGAQIDLKQNDNCTALHFAATQGATEILKLM
Elaphe prasina            VCLEIIIKRGEELGYSPENHINFTNNGKCSPLHLAVQSRDLEMIKMCIEYGAQIDLKQNDNCTALHFAATQGATEILKLM
Pseudoxenodon macrops     LCLEIIIKRGEELGYSPENHINFTNNGKCSPLHLAVQSRDLEMIKMCIEYGAQIDLKQNDNYTALHFAATQGATEILKLM
Amphiesma sp.             LCLEIIIKRGEELGYSPENHINFTNNGKCSPLHLAVQSRDLEMIKMCIEYGAQIDLKQNDNCTALHFAATQGATEILKLM
Xenochrophis piscator     LCLEIIIKRGEELGYSPENHINFTNNGKCSPLHLAVQSRDLEMIKMCIEYGAQIDLKQNDNCTALHFAATQGATEILKLM
Plagiopholis blakewayi    LCLEIIIKRGEELGYSPENHINFTNNGKCSPLHLAVQSRDLEMIKMCIEYGAQIDLKQNDNCTALHFAATQGATEILKLM
Naja atra                 LCLEIIIRRGEELGYSPAKHINFTNNKKCSPLHLAVQSRDLEMIKMCIEYGAQIDLKQSDNCTALHFAATQGATEILKLM
Bungarus multicinctus     LCLEIIIKRGEELGYLPANHINFTNNKKCSPLHLAVQSRDLEMIKMCIEYGAQIDLKQSDNCTALHFAATQGATEILKLM
Enhydris chinensis        LCLEIIIKRGEELGYSPENHINFTNNGKCSPLHLAVQSRDLEMIKMCIEYGAQIDLKQNDNCTALHFAATQGATEILKLM
Pareas margaritophorus    LCLEIIIKRGEELGYSPENHINFTNNGKCSPLHLAVQSRDLEMIKMCIEYGAQIDLKQSDNCTALHFAATQGATEILKLM
Ramphotyphlops braminus   LCLEILMKEGEKHGHSPEELINFTNNGKCSPLHLAVQSGDLEMIKMCIEYGAKIDLKQNE-CTALHYAATQGATEILKLM
Anolis carolinensis       ACMEILIKRGEETGYSPENHINFTNNGKCTALHLAVQSGDLEMIKMCIEYGAQIDLKQNDKCTALHFAATQGATEILKLM
Taeniopygia guttata       ACLEILLKKGEELGHSAKTHINFTNNGKCSPLHLAVQSGDLEMIKMCIEFGAQIDLKQNEKCTALHFAATQGATEIVKLM
Gallus gallus             TCMEILLKKGEELGHSAKTHINFTNNGKCSPLHLAVQSGDLEMIKMCIEFGAQIDLKQNEKCTALHFAATQGATEIVKLM
Bos taurus                KCMEIILKFGEEHGYSRLSHINFVNSGKASPLHMAVQSGDLDMIKMCLDNGAQLDLLEKGKCTALHFAATQGATEIVKLM
Mus musculus              KCMELILAYGEKNGYSRETHINFVNHKKASPLHLAVQSGDLDMIKMCLDNGAHIDMMENAKCMALHFAATQGATDIVKLM
Canis familiaris          KCMEIILKFGEEHGYSRQSHINFVNNGKSSPLHMAVQSGDLEMIKMCLDNGAHLELMENGKCTPLHFAATQGATEIVKLM
Monodelphis domestica     KCLEMLLKFGEEQGYSRQFQMNFINNKNSSPLHFAVQSGDLEVIKICLENGAEINLEETGKCTALHFAATQGATEIVKLM
Xenopus tropicalis        KCMDIILKKGEEMGYTIDHHINFTNNEKSSPLHLAVQNGNIDVVKACISYGAKIDLRQSDNATALHFAATQGATEIVKFM
```

```
                              250          260          270          280          290          300          310          320
                          ....|....|....|....|....|....|....|....|....|....|....|....|....|....|....|....|
Crotalus atrox            MSAYSGEECLINASDENKETLLHRAALFDHDEMTDYLISKGANIDSVDIEGRTPLILATSRASWKIVNLLISKGANIQLK
Protobothrops jerdonii    MSAYTGEECLINAYDGNKETLLHRAALFDHDEMTEYPISKGANIDSVDIEGRTPLLLATSRASWKIVNLLIAKGANIELK
Trimeresurus stejnegeri   LSAYSGEECLINAVDGNKETLLHRAALFDHDEMTEYLISKGANIDSVDIEGRTPLLLATSRASWKIVNMLISKGANIGLK
Ovophis monticola         LSSYSGEECLINASDGNKETLLHRAALFDHDEMTEYLISKGANIDSVDIEGRTPLLLATSRASWKIVNLLISKGANIELK
Gloydius brevicaudus      LSSYSGEECLINALDGNKETLLHRAALFDHDEMTEYLIAKGANIDSVDIEGRTPLLLATSRASWKIVNLLISKGANIEIK
Python regius             ISSYTGEVSLINVVDGKKETLLHRAALFDHCEMAEYLISMGAHIDSVDTEGRTPLLLATSCGSWKLVNLLLSKGANLELK
Python molurus bivittatus MSSYTGEESLINVVDGKKETLLHRAALFDHCEMAEYLISMGANIDSVDTEGRTPLVLATSCASWKIVNLLLSKGANLELK
Corallus hortulanus       MSTYEGDESIVNAADGNKETMLHRAALFDHCEMAEYLISKGAKIDCVDHEGRTPLLLGTACGSWKIVNLLLSKGANLELK
Xenopeltis unicolor       MSSYTGDEPLINALDGNKETLLHRAALFDHCELAEYLISMGANIDSIDIEGRTPLLLATSCASWKIVNLLLSKGANLELK
Eryx tataricus            MSSYAGDEPIINAVDGNKETMLHRAALFDHCELAEYLISKGANIDSVDIEGRTPLLLATSCASWKIVNLLLSKGANLELK
Daboia russelii           MSSYSGEEPIINALDGNKETLLHRAALFDHYELAEHLISKGANIDSVDIEGRTPLLLATSCASWKIVNLLLSKGANLELK
Pantherophis obsoletus    MSSYTGEESIINALDGNKETLLHRAALFDHHELAEYLISKGANINSVDIEGRTPLLLATSCASWKIVNLLLSKGANVELK
Ptyas korros              MSSYTGEESIINALDGNKETLLHKAALFDHCELAEYLISKGANINSVDIEGRTPLLLATSCASWKIVNLLLSKGANVELK
Oligodon lacroixi         MSSYTGEESIINALDGNKETLLHRAALFDHCELAEYLISKGANINSVDVEGRTPLLVATSCASWKIVNLLLSKGANVELK
Elaphe prasina            MSSYTGEESIINALDGNKETLLHRAALFDHYELAEYLISKGANINSVDIEGRTPLLLATSCASWKIVNLLLSKGANVELK
Pseudoxenodon macrops     MLSYKGEESIINALDGNKETLLHRAALFDHCELTEYLISKGANINSVDIEGRTPLLLATSCASWKIVNLLLSKGANVELK
Amphiesma sp.             MSSYTGEESIINALDGNKETLLHRAALFDHCELAEYLISKGANINSVDVEGRTPLLLATSCASWKIVNLLLSKEANVELK
Xenochrophis piscator     MSSYTGEESLINALDGNKETLLHRAALFDHYELTEYLISKGADINSVDVEGRTPLLLATSCASWKIVNLLLSKGANVELK
Plagiopholis blakewayi    MSSYKGEESIINALDGNKETLLHRAALFDHCELTEYLISKGANINSVDIEGRTPLLLATSCASWKIVNLLLSKGANVELK
Naja atra                 MSSYTGEDSIINALDGSKETLLHRAALFDHCELAEYLISKGANIDSVDIEGRTPLLLATSCASWKIVNLLLSKGANVELK
Bungarus multicinctus     MSSYTGEDSIINALDGNKETLLHRAALFDHCELAEYLISKGANIDCVDIEGRTPLLLATSCASWKIVNLLLSKGANVQLK
Enhydris chinensis        MSSYTGEESIINALDGNKETLLHRTALFDHYELAEYLISKGANIDSADIEGRTPLLLATSCASWKVVNLLLSKGANIELK
Pareas margaritophorus    MSSYKGEEPIINALDENKETLLHRAALFDHCELADYLISKGANIDSVDIEGRTPLLLATSCASWKIMNLLLSKGANVELK
Ramphotyphlops braminus   ISSYTGDESIMDALDGNKETLLHRAALFDHSELAEYLISMGINIDSVDVEGRTPLLLATSCESWKIVNLLLSKGANVEIK
Anolis carolinensis       MSSYTGDEPIIDALDENKETLLHRVALFDHYELAEYLITMGAKIDSVDIEGRTPLLLATSCASWKIVNLLLSKGANVELK
Taeniopygia guttata       MSSYAGEESIIDAVDGNKETLLHRTALFDHYELAEYLISMGANIDSVDIEGRSPLLLATSCASWKIVNLLLSKGANVSLK
Gallus gallus             MSSYAGDESIIDAVDGNKETLLHRTALFDHYELAEYLISTGANIDSVDTEGRSPLLLATSCASWKIVNLLLSKGANVSLK
Bos taurus                VSSYSGNSDIVNEVDGNHETLLHRASLFDHHELADYLISVGADIDITDSEGRSPLLLATASASWNTVNLLLSKGARVDIK
Mus musculus              ISSYTGSSDIVNAVDGNQETLLHRASLFDHHDLAEYLISVGADINSTDSEGRSPLILATASASWNIVNLLLCKGAKVDIK
Canis familiaris          ISSYSGSSDIVNAVDGNQETLLHRASLFDHHELADYLISVGADINSTDSEGRSPLILATASASWNIVNLLLSKGARVDIK
Monodelphis domestica     ITSYSGNNDIINSVDGNNETLLHRVALFDHHELAEYLISMGANIDSIDLEGRSPLLLATSSASWNVVNLLLSKGANVEIK
Xenopus tropicalis        VSSYSDDNKIVDLPDGNDETPLHKSVLFDHVELAEYLISEGANIDSEDKESRTPLLLATSCSAWKSVNLLLSKGADVKLK
```

ANK7 ANK8 ANK9

↓ L330M in yokoyama et al. (2011)

Q391H in yokoyama et al. (2011)
S434T in yokoyama et al. (2011)

ANK10 ANK11 ANK12

```
                                  330       340       350       360       370       380       390       400
Crotalus atrox               DHLGRSFLHLTVLHPGGLQHLNEEFLKMKHIRDLLTDEDHEGCTPLHYACKQGMPLTVNILLGMNVSVYAKSRDKKSPLH
Protobothrops jerdonii       DHLGRTFLHLTVLHPGGLQHLTDNILEMKTIRKLLTEEDHEGCTPLHYACKQGMPLSVNLLLDMNVSVYAKSRDKKSPLH
Trimeresurus stejnegeri      DHLGRTFLHLTVLQPGGLKHLGENILEMKKIRDLLTEEDNEGCTPLHYACKQGMPLTANILLDMNVSVYAKSRDKKSPLH
Ovophis monticola            DHLGRTFLHLTVLHAGGLQHLNENFLEMKHIRDLLTEEDHEGCTPLHYACKQGMPLSANILLEMNVSVYAKSRDKKSPLH
Gloydius brevicaudus         DHLGRTFLHLTVLHPGGLQHLNEDFLKMKHIRNLLIEEDHEGCTPLHYACKQGMPLSANILLEMNVSVYAKSRDKKSPLH
Python regius                DHLGRNFLHLTVLHPGGLQHLSEQFLKMKVIEDLVGEEDREGCTPLHYACKQGVPLTVNILLKMNVSVYAKSREKKSPLH
Python molurus bivittatus    DHLGRNFLHLTVLHPGGLQHLSEQFLKMKVIENLVSEEDREGCTPLHYACKQGVPLSVNILLKMNVSVYAKSREKKSPLH
Corallus hortulanus          DHLGRNVLHLAVLHPGGLQRLNEEILKLTCIRNLATEEDHEGCTPLHYASRQGVPLTINTLLSMNVSVYCKSRDKKSPLH
Xenopeltis unicolor          DHLGRNFLHLTVLHPGGLQHLNEHFLKMKRIEDLVTEEDHEGCTPLHYACKQGAPISVNILLEMNVSVYSKSRDKKSPLH
Eryx tataricus               DHLGRNFLHLTVLHPGGLQHLNEEFLKMKDIKDLVTEEDHEGCTPLHYACKQGVPHSVNVLLEMNVSVYSKSRDKKSPLH
Daboia russelii              DHLGRNFLHLTVLHPGGLQHLNEDFLKMKHIKDLITEEDHEGCTPLHYACKQGMPLSVNILLEMNVSVYSKSRDKKSPLH
Pantherophis obsoletus       DLLGHNFLHLTVLQPGGLQHLNEDFLKMKHIRDLITEEDQEGCTPLHYASKQGVPLSVNILLEMNVSVYAKSRDKKSPLH
Ptyas korros                 DLLGHNFLHLTVLQPGGLQHLNEDFLKMKHISDLITEEDQEGCTPLHYACKQGVPLSVNILLEMNVSVYAKSRDKKSPLH
Oligodon lacroixi            DLLGRNFLHLTVLQPGGLQHLNEDFLKMKHISDLITEEDQEGCTPLHYACKQGVPLSVNILLGMNVSVYAKSRDKKSPLH
Elaphe prasina               DLLGRNFLHLTVLQPGGLQHLNEDFFKMKHISDLITEEDQEGCTPLHYACKQGVPLSVNILLEMNVSIYAKSRDKKSPLH
Pseudoxenodon macrops        DLLGRNFLHLTVLQPGGLQHLSEHFLKMKHIKDLITEEDQEGCTPLHYASKQGIPLSVNILLEMNVSVYSKSRDKKSPLH
Amphiesma sp.                DLLGRNFLHLTVLQPGGLQHLNEHFLKMKHIKELLTEEDQEGCTPLHYASKQGVPLSVNILLEMNVSVYSKSRDKKSPLH
Xenochrophis piscator        DLLGRNFLHLTVLQPGGLQHLNEHFFKMKHIKDLLTEEDQEGCTPLHYASKQGVPLSVNILLEMNVSVYSKSRDKKSPLH
Plagiopholis blakewayi       DLLGRNFLHLTVLQPGGLQHLSEHFLKMKHIKDLITEEDQEGCTPLHYASKQGIPLSVNILLEMNVSVYSKSRDKKSPLH
Naja atra                    DLLGRNFLHLTVLQPGGLQHLNEHFLKMKHIEHLLSEEDQEGCTPLHYASKQGIPLSVNILLEMNVSVFSKSRDKKSPLH
Bungarus multicinctus        DLLGRNFLHLTVLQPGGLQHLNEHFLKMKHIEHLITEEDQEGCTPLHYASKQGIPLSVNILLEMNVSVYSKSRDKKSPLH
Enhydris chinensis           DLLGRNFLHLTVLQPGGLQHLNEHFLKMKHIKDLITEEDHEGCTPLHYACKQGIPLSVNILLEMNVSVYSKNRDKKSPLH
Pareas margaritophorus       DHLGRNFLHLTVLHPGGLQHLNEHFLKMKHIKDLITDEDNEGCTPLHYACKQGIPLSVNILLEMNVSVYSKSRDKKSPLH
Ramphotyphlops braminus      DHLGRNFLHLTVMHPGGLQQLNDQFLKMKHIKYLVTEEDHGGCTPLHYACRQGVSLSVNSLLDMNVSIYSKSRDKKSPLH
Anolis carolinensis          DHLGRNFLHLTVLHPGGLKHLNEQFLKMKHIKELVTDEDNDGCTPLHYACRQGVPLSVNSLLELDVSIYSKSRDKKSPLH
Taeniopygia guttata          DHLGRNFLHLTVLQPGGLQHLNEKYLQMEHIKNLVVDEDNEGCTPLHYACRQGVALSVNNLLSLNVSIYSKSRDKKSPLH
Gallus gallus                DHLGRNFLHLTVLQPGGLQHLNEKFLQMEHIKNLVVDEDNEGCTPLHYACRQGVALSVNNLLSLNVSIYSKSRDKKSPLH
Bos taurus                   DNLGRNFLHLTVQQPYGLKNLHPEFLQMQHIKELVMDEDNDGCTPLHYACRQGVPVSVNNLLNFNVSIHSKSKDKKSPLH
Mus musculus                 DHLGRNFLHLTVQQPYGLRNLRPEFMQMQHIKELVMDEDNDGCTPLHYACRQGVPVSVNNLLGFNVSIHSKSKDKKSPLH
Canis familiaris             DNLGRNFLHLTVQQPYGLKNLRPEFMQMQHIKNLVMDEDDDGCTPLHYACRHGIPVSVNNLLDFNVSLRSKSKDKKSPLH
Monodelphis domestica        DHYGRNFLHLTVLQPYGLKNLKQEFLQMKHIENLVMDEDIDGCTPLHYACRQGVALSVNNLLRLNVSIGTKSKDNKSPLH
Xenopus tropicalis           DHYGRNFLHLMVLQPGGLKNLNSEFLQMENIKKLVSDEDCEGCTPLHYACRHGVPNSVNNLLGLNMSLYSKSKNKRSPLH
```

```
                                  410       420       430       440       450       460       470       480
Crotalus atrox               FAASHGRLNTCLRLLESMDDTRLLNEGDRKGMTPLHLAAQYGHEKVTQLLLKKGALFNSDYKGWTPLHHAALGGYSRTME
Protobothrops jerdonii       FAASHGRLNTCLRLLDSMDDTRLLNEGDRKGMTPLHLAAQYGHEKVTQLLLKKGALFNSDYKGWTPLHHAAMGGYSRTMQ
Trimeresurus stejnegeri      FAASHGRLNTCLRLLESMEDTRLLNEGDRKGMTPLHLAAQYGHEKVTQLLLKKGALFNSDYKGWTPLHHAALGGYSRTMQ
Ovophis monticola            FAASHGRLNTCLRLLESMEDTRLLNEGDRKGMTPLHLAAQYGHEKVTQLLLKKGALFNSDYKGWTPLHHAALGGYSRTMQ
Gloydius brevicaudus         FAASHGRLNTCLRLLESMEDTRLLNEGDRKGMTPLHLAAQYGHEKVTQLLLKKGALFNCDYKGWTPLHHAALGGYTRTME
Python regius                YAASYGRIHTCHRLLESMPDTRLLNEGDKKGLTPLHLASQNGHEKVVQLLLKRGALFGCDNKGWTALHHAAFGGYTRTMQ
Python molurus bivittatus    YAASYGRINTCHRLLESMPDTRLLNEGDKKGMTPLHLAAQNGHEKVVQLLLKRGALFGCDNQGWTALHHAAFGGYTRTMQ
Corallus hortulanus          FAASNGRLNTCCLLLEAMKDTRLLNEGDKKGMTPLHLAAENGHEKIAQFLLKKGALFLSDNKGWTALHHAAFGGYSRTMQ
Xenopeltis unicolor          FAASCGRINTCHRLLEAMEDTRLLNEGDKKGMTPFHLAAQNGHDKVVQFLLKKGALFLSDYKGWTALHHAAFGGYTRTIQ
Eryx tataricus               FAASYGRINTCRQLLEAMEDTRLLNEGDKKGMTPLHLAAQNGHEKVVQFLLKKGALFLCDYKGWTALHHAAFGGYTRTMQ
Daboia russelii              FAASYGRINTCLRLLEAMEDSRLLNEGDKKGMTPLHLAAQNGHEKVVQFLLKKGALFLCDYKGWTALHHAALGGYTRTMQ
Pantherophis obsoletus       FAASYGRINTCLRLLEAMEDTRLLNEGDKKGMTPLHLAAQNGHEKVVQFLLKKGALFLCDYKGWTALHHAAFGGYTRTMQ
Ptyas korros                 FAASYGRINTCLRLLEAMEDTRLLNEGDKKGMTPLHLAAQNGHEKVVQFLLKKGALFLCDYKGWTALHHAAFGGYTRTMQ
Oligodon lacroixi            FAASYGRTNTCLRLLEAMEDTRLLNEGDKKGMTPLHLAAQNGHEKVVQFLLKKGALFLCDYKGWTALHHAAFGGYTRTMQ
Elaphe prasina               FAASYGRINTCLRLLEAMEDTRLLNEGDKKGMTPLHLAAQNGHEKVVQFLLKKGALFLCDYKGWTALHHAAFGGYTRTMQ
Pseudoxenodon macrops        FAASYGRINTCFRLLEAVEDTRLLNEGDKKGMTPLHLAAQNGHEKVVQFLLKKGALFLCDYKGWTALHHAAFGGYTRTMQ
Amphiesma sp.                FAASYGRINTCLRLLEAMEDTRLLNEGDKKGMTPLHLAAQNGHEKVVQLLLKKGALFLCDYKGWTALHHAAFGGYTRTMQ
Xenochrophis piscator        FAASYGRINTCLKLLEAMEDTRLLNEGDKKGMTPLHLAAQNGHEKVVQLLLKKGALFLCDYKGWTALHHAAFGGYTRTMQ
Plagiopholis blakewayi       FAANYGRINTCFRLLEAMEDTRLLNEGDKKGMTPLHLAAQNGHEKVVQLLLKKGALFLCDYKGWTALHHAAFGGYTRTMQ
Naja atra                    FAASYGRINTCLRLLEAMEDTRLLNEGDKKGMTPLHLAAQNGHEKVVQFLLKKGAFFLCDYKGWTALHHAAFGGYTRTMQ
Bungarus multicinctus        FAASYGRINTCLRLLEAMEDTRLLNEGDKKGMTPLHLAAQNGHEKVVQFLLKKGALFLCDYKGWTALHHAAFGGYTRTMQ
Enhydris chinensis           FAASYGRINTCLRLLEAMEDTRLLNEGDKKGMTPLHLAAQNGHEKVVQFLLKKGALFLCDYKGWTALHHAAFGGYTRTMQ
Pareas margaritophorus       FAASYGRINTCLRLLEAMEDTRLLNEGDKRGMTPLHLAAQNGHEKVVQFLLKKGALFLCDYKGWTALHHAAFGGYTRTMQ
Ramphotyphlops braminus      FAASFGRINTCHRLVRDMVDTRLLNEGDRKGMTPLHLAAQNGHEKVVQFLLKKGALFLCDNNGWTALHHAAFGGYTRTMQ
Anolis carolinensis          FAASYGRINTCQRLLRDMVDTRLLNEGDKKGMTPLHLAAQNGHEKVVQFLLRKGALFLCDYKGWTALHHAAFGGYTRTMQ
Taeniopygia guttata          FAASYGRINTCQRLIRDMKDTRLLNEGDKKGMTPLHLAAQNGHEKVVQFLLKRGALFLCDYKGWTALHHAAFGGYTRTMQ
Gallus gallus                FAASYGRINTCQRLIRDMKDTRLLNEGDKKGMTPLHLAAQNGHEKVVQFLLKRGALFLCDYKGWTALHHAAFGGYTRTMQ
Bos taurus                   FAASYGRINTCQRLLQDISDTRLLNEGDLHGMTPLHLAAKNGHDKVVQLLLKKGALFLSDHNGWTALHHASLGGYTQTMK
Mus musculus                 FAASYGRINTCQRLLQDISDTRLLNEGDLHGMTPLHLAAKNGHDKVVQLLLKKGALFLSDHNGWTALHHASMGGYTQTMK
Canis familiaris             FAASYGRINTCQRLLQDMSDTRLLNEGDLHGMTPLHLAAKNGHDKVVQLLLKKGALFLSDHNGWTALHHASLGGYTQTMK
Monodelphis domestica        FAANFGRINTCQRLIRDMTDTRLLNEGDQNGMTPLHLAAKNGHDKVVKFLLKKGALFLSDYNGWTALHHASMGGYTQTMK
Xenopus tropicalis           FAACYGRFNTCQRLLRFMSDGRLLNDGDEKGMTPLHLAAENGHEKIVFLLLKRGALLLSDHRGWTALHYAALSGYTRTIK
```

ANK12 ANK13 ANK14

```
                              ANK14          ANK15                        ANK16
                                    490       500       510       520       530       540       550       560
                              ....|....|....|....|....|....|....|....|....|....|....|....|....|....|....|....|
Crotalus atrox                IILNTNMKSTDKVNDKGDTALHLAAREGHARAVKLLLDANAKILLNESEASFLHEAIHNERKDVVKIVILHKRWEESIST
Protobothrops jerdonii        IILNTNMKSTDKVNDKGDTALHLAAREGHARAVKLLLDANAKILLNETEASFLHEAIHNERKEVVKIVILHKRWEESITT
Trimeresurus stejnegeri       ITLNTQMKSTDKLNDKGDTALHLAAREGHARAVKLLLDANAKILLNETDASFLHEAIHNERKEVVKIVILHKRWEESITT
Ovophis monticola             IILNTNMKSTDKVNDKGDTALHLAAREGHARAVKLLLDANAKILLNETDASFLHEAIHNERKDVVKIVILHKRWEESITT
Gloydius brevicaudus          IILNTNMKATDKVNDKGDTALHLAAREGHARAVKLLLDSNAKILLNGTDASFLHEAIHNERKDVVKVVILHKRWEESITT
Python regius                 IILNTNMIATDKEDEDGNTGLHLAAREGHAKAVKLLLDGNAKIVLNKAEASFLHEAIRNGRKNVVNVVILHKRWEESMTT
Python molurus bivittatus     IILNTNMIATDKQDEDGNTGLHLAAREGHAKAVKLLLDGSAKILLNKAEASFLHEAIRNGRKNIVNVVILHKRWEESMST
Corallus hortulanus           AVLDTNVKATDNVDEDGNTALHLAAREGHAKAVKLLLDEGAKILLNKVEASFLHEAIHNGQRDVVTVVILHKRWEEAIST
Xenopeltis unicolor           IILDTNVKATDNEDEEGNTGLHLAAREGHAKAVKLLLDNNAKILLNKVEASFLHEAIRNGQKDVVNVVILHKRWEESMTT
Eryx tataricus                VILETNVKATDNVDEEGNTALHLAAREGHAKAVKLLLDDNAKILLNKAEASFLHEAIHNGQKDVVNVVILHKRWEESVTT
Daboia russelii               IILNTNMKATDKVNEEGNTALHLAAREGHAKAVKLLLDENAKILLNRAEASFLHEAIHNGQKDVVNVVILHKRWEESITT
Pantherophis obsoletus        IILNTNMKATDKVNDEGNTALHLAAREGHAKAVKLLLDDNAKILLNSAEASFLHEAIHNGRKDVVNAVILHKRWEESITT
Ptyas korros                  IILNTNMKATDKVNDEGNTALHLAAREGHAKAVKLLLDDSAKILLNRAEASFLHEAIHNGRKDVVNTVILHKRWEESITT
Oligodon lacroixi             IILNTNMKATDKVNDEGNTALHLAAREGHAEAVKLLLDDSAKIVLNRAEASFLHEAIHNGRKDVVNAVILHKRWEESITT
Elaphe prasina                IILNTNMKATDKVNEEGNTALHLAAREGHAKAVKLLLDDSAKILLNRAEASFLHEAIHNGQKDVVNAVILHKRWEESITT
Pseudoxenodon macrops         IILNTNMKATDKVNDEGNTALHLAAREGHAKAVKLLLDDNAKILLNRAEASFLHEAIHSGRKDVVNAVILHKRWEESITT
Amphiesma sp.                 IILNTNMKATDKVNDEGNTALHLAAREGHAKAVKLLLDDNAKILLNRAEASFLHEAIHSGRKDVVNAVILHKRWEESIIT
Xenochrophis piscator         IILNTNMKATDKVNDEGNTALHLAAREGHAKAVRLLLDDNAKILLNRAEASFLHEAIHSGRKDVVNSVILHKRWEESIIT
Plagiopholis blakewayi        IILNTNMKATDKVNDEGNTALHLAAREGHAKAVKLLLDDNAKILLNRAEASFLHEAIHSGRKDVVNAVILHKRWEESITT
Naja atra                     IILNTNMKITDKVNDEGNTALHLAAREGHAKAVMLLLDDNAKIVLNRAEASFLHEAIHNGRKDVVNATILHKRWEESIIT
Bungarus multicinctus         IILNTNMKITDKVNDEGNTALHLAASEGHAKAVILLLDDNANILLNRAEASFLHEAIHNGRKDVVNAIILHKRWEESIIT
Enhydris chinensis            IILNTNMKATDKVNDEGNTALHLAAREGHAKAVKLLLDDNAKILLNRTEASFLHEAIHNGQRDVVNTVILHKRWEESIVT
Pareas margaritophorus        IILNTNMKATDKVNHEGNTALHLAAREGHAKAVKLLLDDNAKIRLNRAEASFLHEAIHNGQKDVVNVVILHKRWEESIAT
Ramphotyphlops braminus       FILNTNMKTTDKVDKEGNTALHLAAREGHGRAVILLLDDNAKILLNKAESSFLHEAIHNGQTTVVNAVILHKRWEEAMTT
Anolis carolinensis           IILNTNVKCTDKVDEEGNTALHLAAKEGHPKAVRLLLDDNAKILLNKSDASFLHEAIHNGRKEVVNATILHKRWEESMTT
Taeniopygia guttata           IILDTNVKCTDRVDEEGNTALHLAAREGHAKAVRLLLDYGAKILFNKAVASFFHEAIHNRRKDVVSAVILHKRWEEAVVT
Gallus gallus                 IILDTNVKCTDKVDEEGNTALHLAAKEGHAKAVRLLLDYGAKILLNKAVASFFHEAIHNRRKDVVSTVILHKRWEEAVLT
Bos taurus                    VILDTNLKCTDRLDEEGNTALHFAAREGHAKAVSLLLSYDADVVLNKQQASFLHVAIHNKRKEVVLTTIRSKRWGECFKV
Mus musculus                  VILDTNLKCTDRLDEEGNTALHFAAREGHAKAVAMLLSYNADILLNKKQASFLHIALHNKRKEVVLTTIRNKRWDECLQV
Canis familiaris              VILDTNLKCTDLLDEEGNTALHFAAREGHAKAVALLLSYDAAIVLNKQQASFLHVAIHNKRKEVVLTTIKNKRWEECLEV
Monodelphis domestica         IILDTNVKCTDHVDEEGNTALHYAAREGHAKAVALLLDYDAQILMNKQSATFLHDAIHNRRKEVVITTIRSKRWNECLAE
Xenopus tropicalis            TLLETSIGLIDKTDKEQNTAMHLAGREGHAKAVSLLLENGASILLNANGASYIHEAIRFGKKDTVLVSIQSERWEEILQT

                                    570       580       590       600       610       620       630       640
                              ....|....|....|....|....|....|....|....|....|....|....|....|....|....|....|....|
Crotalus atrox                FSHFSSINKCAILEMVEYLPDCLKLVLDNCIVKSPEDKGSKDFFVEYNFTYLQCPLQFKKKVKENEGVVYEPLLALNGMV
Protobothrops jerdonii        FSHFSTMNKCAILEMVEYLPDCLKLVLDNCIVKSPEDKGSKDFFVEYNFTYLQCPLQLKKKVKENEGVVYEPLLALNGMV
Trimeresurus stejnegeri       FSHFSSINKCAILEMVEYLPDCLKLVLDNCIVKSPEDKGSKDFFVEYNFTYLQCPLQFKKKSKDNEGAIYEPLLALNGMV
Ovophis monticola             FSHFSSINKCAILEMVEYLPDCLRLVLDNCIVKSPEDKGSKDFFVEYNFTYLQCPLQLKKKVKENAGAVYEPLLALNGMV
Gloydius brevicaudus          FSHFSSVNKCAILEMVEYLPDCLKLVLDNCIVKSPEEKGSKDFFVEYNFTYLQCPLQLKKKVKENEGAVYEPLLALNGMV
Python regius                 FSHNSSINKCAILEMVEYLPDCLKLVLDNCIVRSPDEKTSRDFSIQYNFRYLQCPLTLTKKSKAQDGVVYEPLLALNAMV
Python molurus bivittatus     FSHHSSVNKCAILEMVEYLPDCLKLVLDNCIVRSPDEKTSRDFSIQYNFRYLQCPLKLTKKSKEHNGIVYEPLLALNAMV
Corallus hortulanus           FSHQSSVNKCAILDMVEYLPGCLKLVLDNCIIRSPEEKTSADFFIEYNFRYLQCPLKLTKKSKGNGGALYEPLLALNAMV
Xenopeltis unicolor           FSHHSSINKCAILEMIEYLPECLKLVLDNCIIKSTDEKASRDFCIEYNFRYLQCPLKLTKKLKECDGVDYEPLLALNAMV
Eryx tataricus                FSHQSSINKCAILEMVEYLPECLKLVLDNCIIQSPDEKTSTDFCIEYNFRYLQCPLKLTKKSKEHDGVIYEPLLALNAMV
Daboia russelii               FSHHSSINKCAILEMVEYLPECLKLVLDNCIIESTDEKGSKEFCIEYNFRYLQCPLKLKKKFKENEGVVYEPLLALNAMV
Pantherophis obsoletus        FSHHSSINKCAILEMVEYLPECLKLVLDNCIIESPDEKGSKDFCIEYNFRYLQCPLKLKKKFKENEGIIYEPLLALNGMV
Ptyas korros                  FSHQSSINKCAILEMVEYLPECLKLVLDNCIIESPDEKGSKDFCIEYNFRYLQCPLKLKKKCKENGGIIYEPLLALNAMV
Oligodon lacroixi             FSHHSSINKCAILEMVEYLPECLKLVLDNCIIESPDEKGSKDFCIEYNFRYLQCPLKLKKKCKENGEIIYEPLLALNAMV
Elaphe prasina                FSHHSSINKCAILEMVEYLPECLKLVLDNCIIESPDEKGSKDFSIEYNFRYLQCPLKLKKKFKENGGIIYEPLLALNAMV
Pseudoxenodon macrops         FSHNSSINKCAILEMVEYLPECLKLVLDNCIVKSPDEKGSKDFCIEYNFRYLQCPLKLKKKFKENGEIIYEPLLALNAMV
Amphiesma sp.                 FSHHSSINKCAILEMVEYIPECLKLVLDNCIIESPDEKGSKDFSIEYNFRYLQCPLKLKKKCKENGGIIYEPLLALNAMV
Xenochrophis piscator         FSHHSSINKCAILEMVEYLPECLKLVLDNCIIESPDEKGSKDFSIEYNFRYLQCPLKLKKKCKENGGIIYEPLLALNAMV
Plagiopholis blakewayi        FSHNSSINKCAILEMVEYLPECLKLVLDNCIVKSPDEKGSKDFCIEYNFRYLQCPLKLKKKFKKNEEIIYEPLLALNAMV
Naja atra                     FSHHSSTNKCAIIEMVEYLPECLKLVLDNCIIESPDEKGSKDFCIEYNFRYLQCPLKLKKKCKENEEIVYEPLVALNAMV
Bungarus multicinctus         FSHHSSSNKCAIIEMVEYLPECLKLVLDNCIIESPDEKGSKDFCIEYNFRYLQCPLKLKKKCKENEEIVYEPLVALNAMV
Enhydris chinensis            FSHYSSINKCAILAMVEYLPECLKLVLDNCIIESPDEKGSKDYCIEYNFRYLQCPLKLQKKCKENGEVVYEPLLALNAMV
Pareas margaritophorus        FSLHSSINKCAILDMVEYLPECLKLVLDNCIIESPDEKGSKDFCIEYNFRYLQCPLKLKKKFKETKESVYEPLIALNAMV
Ramphotyphlops braminus       FSHHSSMNKCAILEMVEYLPECLKLVLDNCITESPEEKTSKDFCIEYNFRYLQCPLKLKKKLLD-DEVDYEPLVALNFMV
Anolis carolinensis           FSHYSSANKCAILEMVEYLPECLKLVLDNCMIESCEEKTCRDFHIEYNFRYLQCPLKLTKQFKEGDEIMYEPLHNLNAMV
Taeniopygia guttata           FSHSSSANKCPLLEMVEYLPDSFKLVLDNCIIESSEEKTSRDFYIEYNFRYLQCPLTLNKKVKDGEDIFYEPLTTLNAMV
Gallus gallus                 FSHYSSANKCPLLEMIEYLPDSFKLVLDNCIIESSEEKTSRDFYIEYNFRYLQCPLALNRKLKEAEDIFYEPLAILNAMV
Bos taurus                    FNHCSPTNRCPVIELVEYLPECMKVLLDFCVMPSTEDKSSQDYHIEYNFTYLQCPLEFTKKGTPVPDIVYEPLLTLNAMV
Mus musculus                  FTHNSPSNRCPIMEMVEYLPECMKVLLDFCMIPSTEDKSCQDYHIEYNFKYLQCPLSMTKKVAPTQDVVYEPLTILNVMV
Canis familiaris              FSHYSPSNGCPVLEMVEYLPECMKVLLDCCIIPSTRDKSCEDYHIEYNFTYLQCPLEFTKKVTPTQDVTYEPLTTLNAMV
Monodelphis domestica         FNTLSVANKCAVLEMVEYLPECMKTLLDICIVESSEERSCQNFHIEYNFKYLQSPVELREN--EVNNTVYGPLTTLNAMV
Xenopus tropicalis            FSHTSS-YKCAILEMIYHLPESFKTLLDRCMTESIGDKKSPDFHVEYNFTYLQCPLSFRKAHKRENNVHYEPLTTLNAMV
                                           ANK17
```

```
                                                                TM1
                                      650       660       670       680       690       700       710       720
                                 ....|....|....|....|....|....|....|....|....|....|....|....|....|....|....|....|
Crotalus atrox                   RHNRVELLSHPVCTQYLLMKWMAYGLRAHILNLAVYSLGLIPLTLLVTSLE-PD-------------ISFD--EPLQ---
Protobothrops jerdonii           RYNRVELLSHPVCTQYLLMKWMAYGLRAHILNLTVYSLGLIPLTLLVTNLE-PE-------------ISFD--EPLQ---
Trimeresurus stejnegeri          RYNRVELLSHPVCTQYLLMKWMAYGLRAHILNLAVYSLGLIPLTLLITNLE-PE-------------ISFD--EPLQ---
Ovophis monticola                RHNRVELLSHPVCTQYLLMKWMAYGLRAHILNLAVYSLGLIPLTLLVTNLE-PD-------------ISFD--EPLQ---
Gloydius brevicaudus             RHNRVELLSHPVCTQYLLMKWMAYGLRAHILNLAVYSLGLIPLTLLVTNLE-PD-------------ISFD--EPLQ---
Python regius                    RYNRMELLSHPVCTEYLLMKWMAYGFRAHIMNLAVYSLGLIPLTLLITSIE-PE-------------VSFN--TTFK---
Python molurus bivittatus        RYNRMELLSHPVCTEYLLMKWMAYGFRAHLMNLAVYSLGLIPLTLLITSIE-PE-------------VSFN--TTFK---
Corallus hortulanus              LHNRMELLSHPVCTEYLLMKWMAYGFRAHIMNLAVYSLGLIPLTLLITSLE-PK-------------VTFN--ATLK---
Xenopeltis unicolor              RYNRVELLSHPVCTEYLLMKWMAYGFRAHIMNLAVYSLGLIPLTILITTIE-PE-------------ASFN--TTLK---
Eryx tataricus                   RHNRVELLSHPVCTEYLLMKWMAYGFRAHLMNLAVYSLGLIPLTILITSLE-PE-------------VSFN--TTVK---
Daboia russelii                  RHNCVELLSHPVCTEYLLMKWMAYGFRAHILNLAVYSLGLIPLTLLITNLE-PH-------------ISFN--ATLQ---
Pantherophis obsoletus           RHNRVELLSHPVCTEYLLMKWMAYGFRAHILNLAVYSLGLIPLTLLVTHLE-PD-------------VCFN--ATLK---
Ptyas korros                     RHNRVELLSHPVCTEYLLMKWMAYGFRAHILNLAVYSLGLIPLTLLVTHLE-PD-------------VCFN--ATLR---
Oligodon lacroixi                RHNRVELLSHRVCTEYLLMKWMAYGFRAHILNLAVYSLGLIPLTILITHLE-PD-------------VCFN--TTLK---
Elaphe prasina                   RHNRVELLSHPVCTEYLLMKWMAYGFRAHILNLAVYSLGLIPLTLLITHLE-PD-------------VCFN--ATLK---
Pseudoxenodon macrops            RHNRVELLSHPICTEYLLMKWMAYGFRAHILNLGVYSLGLIPLTLLVTYLE-PD-------------VSFN--ATLK---
Amphiesma sp.                    RYNRVELLSHPICTEYLLMKWMAYGFRARILNLAVYSLGLIPLTLLITNLE-PD-------------VSFN--ATLK---
Xenochrophis piscator            RHNRVELLSHPVCTEYLLMKWMAYGFRAHILNLAVYSLGLIPLTLLITNLE-PD-------------VSFN--ATLK---
Plagiopholis blakewayi           RHNRVELLSHPISTEYLLMKWMAYGFRAHILNLGVYSLGLIPLTLLVTYLE-PD-------------VSFN--ATLK---
Naja atra                        RHNRVELLSHPISTEYLLMKWMAYGFRAHILNLAVYSLGLIPLTLLITHLE-PD-------------VSFN--ATLK---
Bungarus multicinctus            RHNRVELLRHSISTEYLLMKWMAYGFRAHILNLAVYSLGLIPLTLLITHLE-PD-------------VSFN--ATLK---
Enhydris chinensis               RHNRVELLSHRVCTEYLLMKWMAYGFRAHILNLAVYSLGLIPLTLLITSLE-PD-------------VSFN--ATLK---
Pareas margaritophorus           RHNCVELLSHPVSTEYLLMKWMAYGFRAHILNLAVYSLGLIPLTLLITNLE-PD-------------ISFN--TTLK---
Ramphotyphlops braminus          RHNRVDLLSHPVCTEYLLMKWMAYGFRAHLMNLAVYSLGLIPLTLLISHTE-SE-------------VSFN--KTFK---
Anolis carolinensis              RHNRVELLSHPVCTEYLLMKWMAYGFKAHLMNLAVYSLGLIPLTLLIVHIERPE-------------PPYN--ATVK---
Taeniopygia guttata              RHNRMELLSHPVCKEYLLMKWMAYGFRAHLMNLGIYSLGLIPLTLLVTHIQ-PG-------------RPLNGTELYE---
Gallus gallus                    RHNRMELLSHPVCKEYLLMKWMAYGFRAHLMNLGIYSLGLIPLTLLVTHIQ-PG-------------RPLNGTEIYE---
Bos taurus                       QHNRIELLNHPVCKEYLLMKWLAYGFRAHVLNLGSYCLGLFPMTFLVVSIR-PG-------------MAFNSTGIIN-ET
Mus musculus                     QHNRIELLNHPVCREYLLMKWCAYGFRAHMMNLGSYCLGLIPMTLLVVKIQ-PG-------------MAFNSTGIINGTS
Canis familiaris                 QHNRIELLNHPVCKEYLLMKWLAYGFRAHIMNLGSYCLGLLPMTFLVVSIK-PG-------------MAFNSTGIIN-ET
Monodelphis domestica            LHNRRDLLDHPVCREYLLMKWMAYGFRAHMMNLGLYCLGLIPMTILISNMQ-PV-------------VAFNSTENIN-ST
Xenopus tropicalis               QHQRIDLLSHPVCKEYLLMKWMAYGFKAHLLNLAIYSLGLIPLTLLIINAVRPESLAIIEECEHLKRTDFYN-SSMKQEC

                                      730       740       750       760       770       780       790       800
                                 ....|....|....|....|....|....|....|....|....|....|....|....|....|....|....|....|
Crotalus atrox                   ---YGPFDNK--------KSYFIKVCMSLVFIMSLFGICKEIIQLFQQKLNYLMDYSNLLDWAIYTTSLIFVSSLLVT--
Protobothrops jerdonii           ---HGPFDNK--------KSYFIKVCMSLVFIMSLFGICKEIIQLFQQKLSYLMDYSNLLDWAIYTTSIIFVSSLLVT--
Trimeresurus stejnegeri          ---HGPFDNK--------KSYFIKVCMSLVFIMSLFGICKEIIQLFQQKLSYLMDYSNLLDWAIYTTSIIFVSSLIVT--
Ovophis monticola                ---HGPFDNK--------KSYFIKVCMSLVFIMSLFGICKEIMQLFQQKLSYLMDYSNLLDWAIYTTSIIFVSSLLVT--
Gloydius brevicaudus             ---YGPFDNK--------KSYFIKVCMSLVFIMSLFGICKEIIQLFQQRLNYLMDYSNLLDWTIYTTSIIFVSSLLVT--
Python regius                    ---HGPFDKK--------DSNFIKVCMCLVFIMSLFGICKEFMQLFQQKLRYLLDYTNLLDWTIYTTSIIFVSSLFVK--
Python molurus bivittatus        ---NGPFDKK--------DSNFIKVCMCLVFIMSLFGICKELMQLFQQKLRYLLDYSNLLDWTIYTTSIIFVSSLFVK--
Corallus hortulanus              ---HGPFDKK--------NSSFIKVCMCLVFIMSLFGICKEIIQLFQQRLKYLLDYSNLLDWTIYTTSIIFVSSLFVK--
Xenopeltis unicolor              ---YGPFDKK--------DSNFIKVCMCLVFIMSLFGICKELIQLFQQKLKYLLDYSNLLDWTIYTTSIIFVSSLFVK--
Eryx tataricus                   ---YGPFDKK--------DSNFIKVCMCLVFIMSLFGICKEIIQLFQQKLKYLLDYSNLLDWTIYTTSIIFVSSLFVK--
Daboia russelii                  ---YGPFDNK--------NSNFIKVCMSLVFIMSLFGICKEIIQLFQQKLNYLLDYSNLLDWTIYTTSIIFVSSLFVT--
Pantherophis obsoletus           ---YGPFDNK--------DSNFIKVCMSLVFIMSLFGICKEIIQLFQQKLNYLLDYSNLLDWTIYTTSIIFVSSLFVM--
Ptyas korros                     ---YGPFDNK--------DSNFIKVCMSLVFIMSLFGICKEIIQLFQQKLNYLLDYSNLLDWTIYTTSIIFVSSLFVM--
Oligodon lacroixi                ---SGPFDNK--------DSSFIKVCMSLVFIMSLFGICKEIIQLFQQKLNYLLDYSNLLDWTIYTTSIIFVSSLFVM--
Elaphe prasina                   ---YGPFDNK--------DSNFIKVCMSLVFIMSLFGICKELIQLFQQKLNYLLDYSNLLDWTIYTTSIIFVSSLFVM--
Pseudoxenodon macrops            ---YGPFDNK--------DSNFIKVCMSLVFIMSLFGICKEIIQLFQQKLNYLMDYSNLLDWTIYTTSIIFVSSLFGM--
Amphiesma sp.                    ---YGPFDNK--------DSNFIKVCMSFVFIMSLFGICKEIIQLFQQKVNYLLDYSNLLDWTIYTTSIIFVSSLFVT--
Xenochrophis piscator            ---YGPFDNK--------DSNFIKVCMSFVFIMSLFGICKEIIQLVQQKLNYLLDYSNLLDWTIYSTSIIFVSSLFVT--
Plagiopholis blakewayi           ---YGPFDNK--------DSNFIKVCMSLVFIMSLFGICKEIIQIFQQKLNYLLDYSNLLDWTIYTTSIIFVSSLFGM--
Naja atra                        ---YGPFDNK--------DSNFIKVCMSLVFIMSLFGICKEIIQLFQQKLNYLLDYSNLLDWTIYTTSIIFVSSLFVM--
Bungarus multicinctus            ---YGPFDNK--------DSNFIKVCMSLVFIMSLFGICKEIIQLFQQKLNYLLDYANLLDWTIYTTSIIFVSSLFVT--
Enhydris chinensis               ---YGPFDNK--------DSNFIKVCMSLLFIMSLFGICKEIVQLFQQKLNYLLDYSNLLDWTIYTTSIIFVSSLFVT--
Pareas margaritophorus           ---YGPFDNK--------DSHFIKVCMSLVFIMSLFGICKEIIQLFQQKLSYLLDYSNLLDWTIYTTSIIFVSSLFVR--
Ramphotyphlops braminus          ---FGPFEKK--------NSYLTKVCMCLVFIMSLFGVLKEIIQLFQQKLKYLLDYSNLLDWTIYTTSMIFVSSLFIK--
Anolis carolinensis              ---SGPFGKKEDRMEININSYFIKVCMCLVFIMSVFGICKEIVQLFQQKLRYLLDYSNLLDWIIYTTSITFVSSLFIN--
Taeniopygia guttata              ---ARPLEYE----VLLLDSYFTRVCMCLVLIMSLLGICKEIFQLIQQKLKYLLDYSNLLDWTIYTTSIIFVSSLFIN--
Gallus gallus                    ---ARPLEYEEER-VPLTDSYFTRVCMCLVLIMSLLGICKEIFQLIQQKLKYLLDYSNLLDWTIYSTSIIFVSSLFIN--
Bos taurus                       SDHSEILDTK--------NSFAINFCMILVLLSSILGYCKEVAQIFQQKRNYFLDKDNAAEWIIYTTSIIFVSPLFIR--
Mus musculus                     STHEERIDTL--------NSFPIKICMILVFLSSIFGYCKEVIQIFQQKRNYFLDYNNALEWVIYTTSIIFVLPLFLN--
Canis familiaris                 TDHSEILDTK--------NSYSIKVCMILVFLSSIFGYCKEVTQIFQQKRNYMLDQANAVEWIIYTTSIIFVLPLFIG--
Monodelphis domestica            NGESKPLDHK--------NSYLIKVCMILVFLSSVFGFCKEMIQIYQQKTNYFLDHTNLIEWVIYTTSIVFVLPLFVS--
Xenopus tropicalis               ANVSRKLDLLQLK-----DTQYTRVCMGLVFCMSLFGIVKEAVQLCQQKLAYIGDGSNIIDWSIYISSIIFVSSLCHSPI
                                                                TM2                           TM3
```

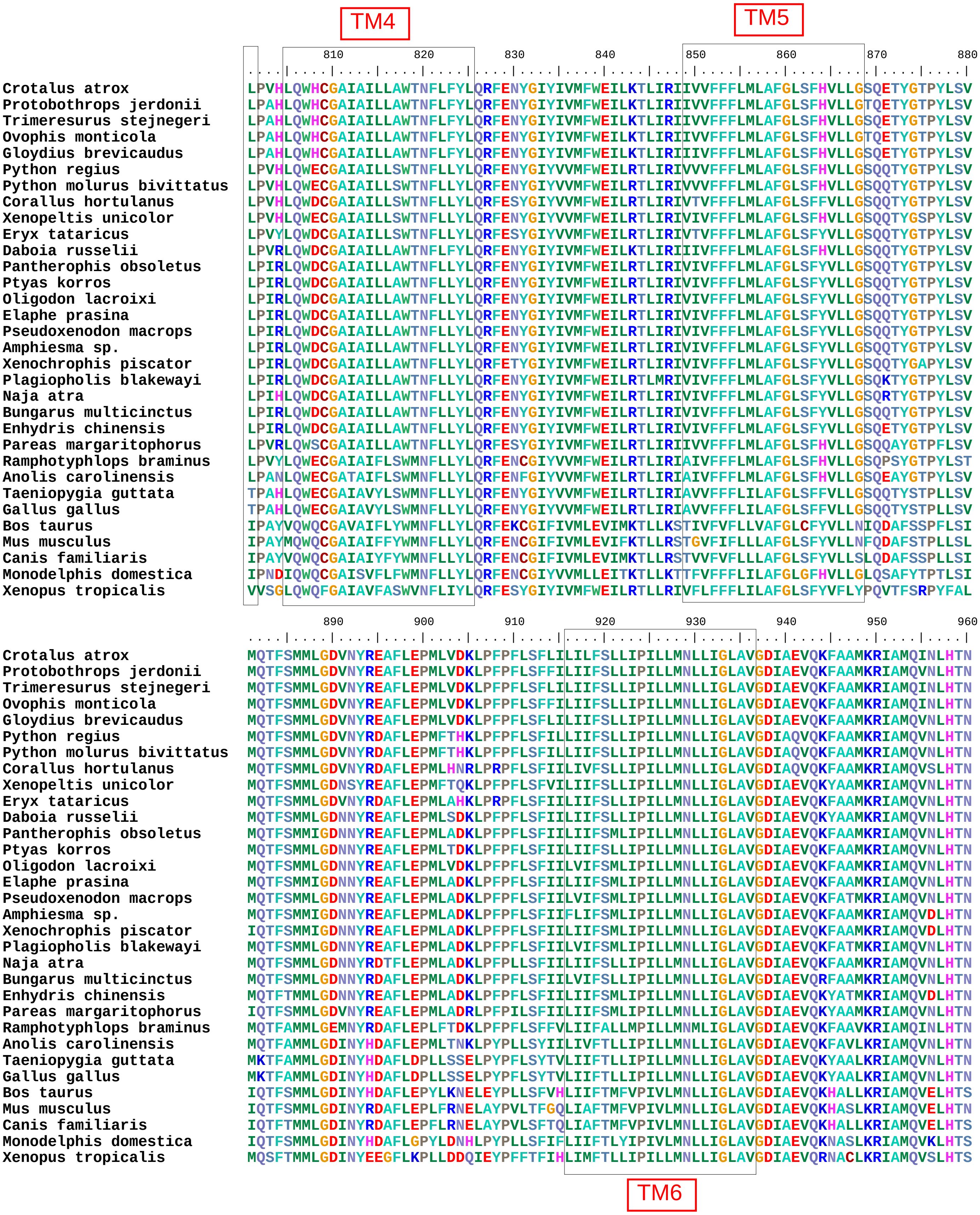

TM4
TM5
TM6
Crotalus atrox
Protobothrops jerdonii
Trimeresurus stejnegeri
Ovophis monticola
Gloydius brevicaudus
Python regius
Python molurus bivittatus
Corallus hortulanus
Xenopeltis unicolor
Eryx tataricus
Daboia russelii
Pantherophis obsoletus
Ptyas korros
Oligodon lacroixi
Elaphe prasina
Pseudoxenodon macrops
Amphiesma sp.
Xenochrophis piscator
Plagiopholis blakewayi
Naja atra
Bungarus multicinctus
Enhydris chinensis
Pareas margaritophorus
Ramphotyphlops braminus
Anolis carolinensis
Taeniopygia guttata
Gallus gallus
Bos taurus
Mus musculus
Canis familiaris
Monodelphis domestica
Xenopus tropicalis

```
                                    970       980       990      1000      1010      1020      1030      1040
                             ....|....|....|....|....|....|....|....|....|....|....|....|....|....|....|....|
Crotalus atrox               LEKKLPYWFLNRVDQQSMVIYPNRS-RFG-GVMS-MFQYCFGWDNIASDAKNADAAIELELLKQKYRLKDTANLVEKQHD
Protobothrops jerdonii       LEKKLPFWFLSRVDQESIVIYPNRS-RYG-GVMS-VFQLCFGWDNITGDAKNADAPLELELLKQKYRLKDTANLVEKQHA
Trimeresurus stejnegeri      LEKKLPFWFLSRVDQQSIVIYPNRS-RYG-GVMS-VFQYCFGWDNIGGDAKYADKPLELELLKQKYRLKDTANLVEKQHT
Ovophis monticola            LEKKLPYWFLNRVDQQSIVIYPNRS-RYG-GVMS-VFQYCFGWDNLADGAKNAEATLELELLKQKYRLKDTANLVEKQHN
Gloydius brevicaudus         LEKKLPYWFLNRVDQQSIVIYPNRS-RYG-GVMS-VFQYCFGWDNIAADSKNAEATLELELLKQKYRLKDTSTLVEKQHN
Python regius                LEKKLPYWFLSRVDQESIILYPNRP-RYC-GFMS-VFHYCFGWDSIAADAQGADTALELEVLKQKYRLKDVALLMEKQHD
Python molurus bivittatus    LEKKLPYWFLSRVDQESIILYPNRP-RYC-GFMS-VFHYCFGWDSIAADTQSADTTLELEVLKQKYRIKDIAALMEKQHD
Corallus hortulanus          LEKKLPYWFLTRVDQETLTLYPNKP-RYC-GFMS-VLNYCFGWDSIAADTQNADTTLELEILKLKYRLKDMATVIEKQHD
Xenopeltis unicolor          LEKKLPYWFLSKVDQESLTVYPNRP-RYC-GFMS-VFQYCFGWDNTAAETQCADTTLELEILKQKYRLKDVAAMMEKQHD
Eryx tataricus               LEKKLPYWFLSRVDQESLILYPNKP-RYC-GFMS-VFHYCFGWDSTAADTQNADTTLELEVLKQKYRLKDIASLIEKQHN
Daboia russelii              LEKKLPYWFLSRVDQQSILVYPNRS-RYC-GFMS-VFQYCFGWDNIAADTKSADTTLELEVLKQKYRLKDIAALVEKQHN
Pantherophis obsoletus       LEKKLPYWFLSRVDQESIVVYPNKP-RYC-GFMT-VFQYCFGWDNTAADTQSADTTLELEVLKQKYRLKDIAALVEKQHN
Ptyas korros                 LEKKLPYWFLSRVDQESIVVYPNRP-RYC-GFMT-VFQYCFGWDNTAADTQSADTTLELEVLKQKYRLKDIATLVEKQHN
Oligodon lacroixi            LEKKLPYWFLSRVDQESIVVYPNRP-RYC-GFMT-VFQYCFGWDNMTADPESADTTLELEVLKQKYRLKDIATLVEKQHN
Elaphe prasina               LEKKLPYWFLSRVDQESIVVYPNKP-RYC-GFMT-VFQYCFGWDNTASDTQSADTTLELEVLKQKYRLKDIATLVEKQHN
Pseudoxenodon macrops        LEKKLPYWFLSRVDQESIVVYPNRP-RYC-GFMS-VFHYCFGWDNTAADTQSADTTLELEVLKQKYRLKDIASLVEKQHN
Amphiesma sp.                LEKKLPYWFLSRVDQESIVLYPNRP-RYC-GFMS-VFHYCFGWDNTAADTQSADITLELEVLRQKYRLKDMATLVEKQHN
Xenochrophis piscator        LEKKLPYWFLSRVDQESIVIYPNRP-RYC-GFMS-VFHYCFGWDNTAADTQSADTTLELEILKQKYRLKDIATLVEKQHN
Plagiopholis blakewayi       LEKKLPYWFLSRVDQESIVVYPNRP-RYC-GFMT-VFHYCFGWDNTAADTQSADTTLELEVLKQKYRLKDIATLVEKQHN
Naja atra                    LEKKLPYWFLSRVDQESIVVYPNRP-RYC-GFMS-VFQYCFGWDNTAADTQSADTTLELEVLKQKYRLKDIATLVEKQHN
Bungarus multicinctus        LEKKLPYWFLSRVDQESIVVYPNRP-RYC-GFMS-VFQYCFGWDNTAADTQNADTTLELEVLKQKYRLKDIATLVEKQHN
Enhydris chinensis           LEKKLPYWFLSRVDQESIVLYPNRP-RYC-GFMS-VFHYCFGWENTAADTQSADTTLELEVLKQKYRLKDIATLVEKQHS
Pareas margaritophorus       LEKKLPYWFLSRVDQESIVIYPNRP-RYC-GFMS-VFHYCFGWDNTAGDIRSADTTLELEVLKQKYRLKDIATLVEKQHN
Ramphotyphlops braminus      LEKKLPYWYLSSVDQDSITVYPNRP-RYC-GFMS-VFQYCFGWDSKRAAKQGADKTLELEVMKMKYRLKDISTLMDKQHS
Anolis carolinensis          LEKKLPYWFLSRVDQESITVYPNRP-RYC-GFMS-VFQYCFGCDDRASNVPSADTSLELEVLKQKYRLKDIATLMEKQHD
Taeniopygia guttata          LEKKLPFWFLSRVDQESITVYPNRP-RYC-GFMS-VFQYCFGCEDSTTYAQSPDTTLELEVLKQKYRLKDISTLLEKQHD
Gallus gallus                LEKKLPFWFLSRVDQESITVYPNRP-RYC-GFMS-VFQYCFGCEDSTTDAQSTDTTLELEVLKQKYRLKDISTLLEKQHD
Bos taurus                   LEKKLPLWFLHKVDQKSIIVYPNRP-RYSRGILRGVFHYIFCPHETRQEIPNVDTSLETEIMKQKYRLKDLSSLLEKQHE
Mus musculus                 LEKKLPLWYLRKVDQRSTIVYPNRP-RHG-RMLR-FFHYFLNMQETRQEVPNIDTCLEMEILKQKYRLKDLTSLLEKQHE
Canis familiaris             LEKKLPLWFLRKVDQKSIVVYPNRP-RCT-GLLR-IFHFIFYTKEARQEIPNVDTSLEIEISKQKYRLKDLTSLLEKQHE
Monodelphis domestica        LEKKLPFWFLHKVDQETIIVYPNR--RKNFGLMN-IFNFFFCARYTKPEIQSADTPLEVEVLKQKYRLKDLAFLLEKQHE
Xenopus tropicalis           LEKKLPFWFLKRVDKISITVYPNRPRIWGITFTSNFFHYFSSCDDVKAEIPINDETMELELWKQKNRLKDLTTMMQKQND

                                   1050      1060      1070
                             ....|....|....|....|....|....|....|....
Crotalus atrox               LLKQIAKKMEVISVVEDEDLNDSLQSQFRKEH-FENTNN
Protobothrops jerdonii       LLKLVAKKMEVISVVEDEDLNDSFQNQFRKEH-LENTNS
Trimeresurus stejnegeri      LLKLVAKKLEVISVVEDEDLNDSFQNQFRKEH-LENTNS
Ovophis monticola            LLKLVAKKLEVISVVEDEDLNDSFQNQFRKEH-LENTNN
Gloydius brevicaudus         LLKLVAKKLEVISVVEDEDQNDSFQNQFRKEH-LENTNT
Python regius                LLKLLTRKLEIISEVEDEDAADTFQQEFRKER-SERINN
Python molurus bivittatus    LLKLLTRRMEIISEVEDEDTADTFQQEFRKER-LERINN
Corallus hortulanus          LLKLVARKLEIISELEDEDLSDLSQANFRREQ-SDRNNS
Xenopeltis unicolor          LLKLVAQKVGIMSEAEDEDSSDLFQHKFRKEQ-LERNNG
Eryx tataricus               LLKLVAQKLEIMSEAEDEDSNDLFQQNFRKEQ-FERNNS
Daboia russelii              LLKLVAHKMEVIFEAEDGDPNDSFQNKFRKEK-LEHKNN
Pantherophis obsoletus       LLKLVAQKMEIMSEAEDEDPNDLFQNKFRKEQ-LEHKNS
Ptyas korros                 LLKLVAQKMEIMSEAEDEDPNDLFQNKFRKEQ-LEHKNS
Oligodon lacroixi            LLKLVAQKMEIMSEAEDEDPNDLFQNKFRKEQ-LEHKNS
Elaphe prasina               LLKLVAQKMEIMSEAEDEDPNDSFQNKFRKEQ-LEHKNS
Pseudoxenodon macrops        LLKLVAQKMEIMSEAEDEHPNDLFQNKFRKEH-LEHRNN
Amphiesma sp.                LIKLVAQKMEIMSEVEDEDPNDLFQNKFRKEQ-LEHKNS
Xenochrophis piscator        LLKLLAQKMEIMSEVEDEDPNDLFQNKFRKEQ-LEHKNS
Plagiopholis blakewayi       LLKLVAQKMEIMSEAEDEHPNDLFQNKFRKEH-LEHRNN
Naja atra                    LLKLVAQKMEIISEAEDEDPSDLFQNKFRKER-LEHKSR
Bungarus multicinctus        LLKLVAQKMEIISEAEDEDPNDLFQNKFRKEQ-LEHKNS
Enhydris chinensis           LLKLVAQKMEVMSEAEDEDPNDLFQNKFRKEQ-LEHKNS
Pareas margaritophorus       LLKLVAQKMEIMSEAEDEDSNDLFQNKFRKEQ-LEHKNS
Ramphotyphlops braminus      LLKLLAGKMEIISEAEDEDSNDLFQHEFRKQQ-MEKDWI
Anolis carolinensis          LLKLVVQKMEIISEAEDEDTNDLFQHKFRKQQ-LEHKNS
Taeniopygia guttata          LIKLIIQKMEIVSEAEDEDSNDLFQHKFRKRQ-LEHKNS
Gallus gallus                LFKLIIQKMEIVSEAEDEDSNDLFQHKFRKRQ-LEHRNS
Bos taurus                   LIKLIIQKMEIISETEDEDYHSSFQDRFKKQQ-LEQRNS
Mus musculus                 LIKLIIQKMEIISETEDEDNHCSFQDRFKKER-LEQMHS
Canis familiaris             LIKLIIQKMEIISETEDEDNHSSFQDRFKKEQ-LEQRNS
Monodelphis domestica        LIKLIIQKMEIISEAEDEDNNFSFQQRFRKQQ-LE-RNS
Xenopus tropicalis           LIKLIIQKMEIVSEAEDEDGDNICQSKMMKKQKLDRKES
```
